# Glucagon signaling via supraphysiologic GCGR can reduce cell viability without stimulating gluconeogenic gene expression in liver cancer cells

**DOI:** 10.1186/s40170-022-00280-1

**Published:** 2022-02-05

**Authors:** Jason Godfrey, Romain Riscal, Nicolas Skuli, M. Celeste Simon

**Affiliations:** 1 Department of Cell and Developmental Biology, Perelman School of Medicine, University of Pennsylvania, Philadelphia, USA; 2 Cancer Biology Graduate Program, Perelman School of Medicine, University of Pennsylvania, Philadelphia, USA; 3 Stem Cell and Xenograft Core, Perelman School of Medicine, University of Pennsylvania, Philadelphia, USA

**Keywords:** Glucagon, GCGR, cAMP, PKA, CREB, Gluconeogenesis, SNU398 cells, Liver cancer

## Abstract

**Background:**

Deregulated glucose metabolism is a critical component of cancer growth and survival, clinically evident via FDG-PET imaging of enhanced glucose uptake in tumor nodules. Tumor cells utilize glucose in a variety of interconnected biochemical pathways to generate energy, anabolic precursors, and other metabolites necessary for growth. Glucagon-stimulated gluconeogenesis opposes glycolysis, potentially representing a pathway-specific strategy for targeting glucose metabolism in tumor cells. Here, we test the hypothesis of whether glucagon signaling can activate gluconeogenesis to reduce tumor proliferation in models of liver cancer.

**Methods:**

The glucagon receptor, GCGR, was overexpressed in liver cancer cell lines consisting of a range of etiologies and genetic backgrounds. Glucagon signaling transduction was measured by cAMP ELISAs, western blots of phosphorylated PKA substrates, and qPCRs of relative mRNA expression of multiple gluconeogenic enzymes. Lastly, cell proliferation and apoptosis assays were performed to quantify the biological effect of glucagon/GCGR stimulation.

**Results:**

Signaling analyses in SNU398 GCGR cells treated with glucagon revealed an increase in cAMP abundance and phosphorylation of downstream PKA substrates, including CREB. qPCR data indicated that none of the three major gluconeogenic genes, *G6PC*, *FBP1*, or *PCK1*, exhibit significantly higher mRNA levels in SNU398 GCGR cells when treated with glucagon; however, this could be partially increased with epigenetic inhibitors. In glucagon-treated SNU398 GCGR cells, flow cytometry analyses of apoptotic markers and growth assays reproducibly measured statistically significant reductions in cell viability. Finally, proliferation experiments employing siCREB inhibition showed no reversal of cell death in SNU398 GCGR cells treated with glucagon, indicating the effects of glucagon in this setting are independent of CREB.

**Conclusions:**

For the first time, we report a potential tumor suppressive role for glucagon/GCGR in liver cancer. Specifically, we identified a novel cell line-specific phenotype, whereby glucagon signaling can induce apoptosis via an undetermined mechanism. Future studies should explore the potential effects of glucagon in diabetic liver cancer patients.

**Supplementary Information:**

The online version contains supplementary material available at 10.1186/s40170-022-00280-1.

## Background

Tumors are often diagnostically distinguished from surrounding normal tissue due to their enhanced uptake of glucose, commonly observed via fluorodeoxyglucose PET imaging [1]. Oncogenic drivers and mitogenic pathways, such as Ras, PI3K/Akt, and HIFs increase the surface expression of glucose transporters and ultimately contribute to its elevated uptake from external sources [2]. Although recent studies suggest intratumoral macrophages may account for most of the glucose consumption [3], elevated glucose catabolism is nonetheless commonly measured in tumors, such as in patient lung cancers [4] and *in vivo* models of liver cancer [5]. Whereas normal somatic cells mainly utilize glucose for energy homeostasis, enhanced glycolytic flux in tumor cells produces anabolic metabolites critically necessary for sustained proliferation [6]. Therefore, targeting glycolysis and disrupting glucose catabolism with pharmacological agents is a rational approach to specifically delay cancer progression [7]. One possibility would be by increasing rates of gluconeogenesis (i.e., glucose production), the opposite biochemical reactions of glycolysis.

Normally, hepatic gluconeogenesis rates are elevated in response to glucagon, a small peptide hormone involved in blood glucose homeostasis [8]. Glucagon is secreted by exocrine pancreatic α-cells in response to a complex network of stimuli [9], including low blood sugar [10]. Glucagon primarily acts on the liver to raise circulating glucose levels by enhancing glycogenolysis [11], lipolysis, fatty acid β-oxidation, and amino acid uptake; the latter three providing carbon and energy necessary for *de novo* glucose synthesis [12, 13]. Mechanistically, glucagon binds to a G-coupled glucagon receptor (GCGR), leading to the generation and intracellular release of secondary messengers cyclic AMP (cAMP) and calcium (Ca2+), respectively [14, 15]. Canonically, cAMP and cytosolic Ca2+ facilitate the auto-phosphorylation of protein kinase A (PKA) and Ca2+/calmodulin-dependent protein kinase II (CAMKII). Previous studies revealed a wide array of targets for PKA and CAMKII, including the transcription factors cAMP response element-binding protein (CREB) and forkhead box protein O1 (FOXO1) [16, 17], that mediate the transcription of glucagon-responsive genes. Increased gluconeogenic gene expression rewires hepatic metabolism to favor the synthesis and export of glucose from non-carbohydrate precursors (i.e., amino acids and fatty acids) in an energy-demanding process [18]. In effect, sustained glucagon signaling regulates blood sugar homeostasis via liver-dependent gluconeogenesis. However, it is unclear whether glucagon signaling is critical in liver cancer cells or whether full activation of gluconeogenesis could antagonize pro-tumorigenic glycolysis.

Three irreversible gluconeogenic reactions are required for full pathway engagement: those regulated by phosphoenolpyruvate carboxykinase (PCK), fructose-1,6-bisphosphatase (FBP), and glucose-6-phosphate catalytic subunit C (G6PC). All of these metabolic enzymes are under investigation in cancer [19]. We previously identified both catalytic and non-catalytic mechanisms of FBP1-dependent tumor suppression in clear cell renal cell and hepatocellular carcinomas [20, 21], and a tumor suppressive role for FBP1 has been noted for pancreatic adenocarcinoma [22], ER-positive breast cancer [23], and gastric cancer [24]. PCK1 overexpression accelerates colorectal xenograft growth [25] but also antagonizes hepatoma proliferation [26], suggesting contextual roles of PCK1 for a given tumor type and metabolic stress, and G6PC protects glioblastoma cells from 2-deoxyglucose (2DG) treatment [27].

The clinical focus of gluconeogenic regulators, glucagon and GCGR, predominantly centers on diabetes, whereby unabated glucagon signaling, due to insulin resistance, contributes to hyperglycemia. In fact, glycemic normalization induced by novel GCGR antagonists are under active assessment in clinical trials [28]. We therefore proposed the following question: can glucagon signaling induce gluconeogenic gene expression to perturb liver cancer? Since gluconeogenesis biochemically opposes glucose catabolism, we hypothesized that glucagon signaling would have a tumor suppressive effect in liver cancer cells that are responsive to glucagon, especially if they express sufficient levels of GCGR and key gluconeogenic enzymes. However, whereas our findings support the anti-tumorigenic role of glucagon and GCGR in one model of liver cancer, we were unable to detect increases in gluconeogenic gene expression. We conclude that glucagon signaling has the potential to oppose cancer growth but may not represent a clinically translatable option at this time.

## Methods

### Cell lines and culture

The following liver cancer cell lines were purchased from the American Type Culture Collection (ATCC): SNU182 (hepatocellular carcinoma, P53^S215I/S215I^, catalog # CRL-2235), SNU387 (hepatocellular carcinoma, NRAS^Q61K/+^, P53^K164*/K164*^, catalog # CRL-2237), SNU398 (hepatocellular carcinoma, β-catenin^S37C/+^, catalog # CRL-2233), SNU423 (hepatocellular carcinoma, P53 splice site mutation, catalog # CRL-2238), SNU449 (hepatocellular carcinoma, P53^A161T/A161T^, catalog # CRL-2234), SNU475 (hepatocellular carcinoma, P53^N239D,G262D/+^, catalog # CRL-2236), HepG2 (hepatoblastoma, NRAS^Q61L/+^, catalog # HB-8065), Hep3B (hepatocellular carcinoma, Axin1^R146*/R146*^, catalog # HB-8064), PLC (hepatoma, P53^R249S/R249S^, catalog # CRL-8024), and SKHEP1 (hepatocellular carcinoma, BRAF^V600E/+^, catalog # HTB-52). Huh7 (hepatoma, P53^Y220C/Y220C^) was a gift from Dr. Terence Gade. Top oncogene hotspot mutations listed for each cell line were compiled from the Broad Institute Cancer Cell Line Encyclopedia (https://portals.broadinstitute.org/ccle). The patient-derived xenograft cell line, M7571, was a gift from Drs. Terence Gade and Katy Wellen. 293T cells were purchased from the ATCC (catalog # CRL-3216).

All cancer cell lines were maintained in RPMI 1640 medium (Gibco, catalog # 21875034) with 10% fetal bovine serum (Gemini Bio, catalog # 900-108), 1% penicillin/streptomycin (Gibco, catalog # 15140122), and incubated at 21% oxygen/5% carbon dioxide. These cells were passaged by aspirating media, washing with DPBS (Corning, catalog # 21-031-CM), detached from plates with 0.25% trypsin-EDTA (Gibco, catalog # 25200056), and re-plated with fresh 10% FBS-containing RPMI media. Most experiments were performed at 5% FBS. For glucose dependent growth assays, cells were cultured in RPMI medium without glucose (Gibco, catalog # 11879020) with dialyzed FBS (Gemini Bio, catalog # 100-108). For lipid dependent growth assays, cells were cultured with delipidated FBS (Gemini Bio, catalog # 900-123). Primary human hepatocytes (PHH) obtained from Life Technologies (catalog # HMCS15) and directly lysed for RNA and protein analysis only without prior culturing. Cryoplateable primary human hepatocytes (cPHH) were obtained from Sigma-Aldrich (discontinued, catalog # MTOXH1000), thawed in Human Hepatocyte Thawing Medium from Sigma-Aldrich, (discontinued, catalog # MED-HHTM), and cultured in Human Hepatocyte Culture Medium from Sigma-Aldrich (discontinued, catalog # MED-HHCM) on collagen-coated plates. THLE3 cells (ATCC, catalog # CRL-11233) were seeded on plates coated overnight with 0.03mg/ml bovine collagen type I (Life Technologies, catalog #A1064401), 0.01 mg/ml bovine fibronectin (Sigma-Aldrich, catalog # F1141), and 0.01 mg/ml BSA (Sigma-Aldrich, catalog # A9576) in modified BEGM medium (Lonza, catalog # CC-3171) without gentamycin, amphotericin, or epinephrine and with an extra 5 ng/ml EGF (Corning, catalog # CB-40052), 70 ng/μl p-ethanolamine, 1% PenStrep, and 10% FBS.

### Compounds

Hormones and inhibitors used in culture for in vitro experiments were as follows: Glucagon (Sigma-Aldrich, catalog #G2044) was prepared in 0.05 M acetic acid (Sigma-Aldrich, catalog # 6283) at a concentration of 1 mg/ml. Forskolin (MedChem Express, catalog # HY-15371) was prepared in DMSO (Sigma-Aldrich, catalog # 2650) at a stock concentration of 10 mM. 666–15 (Sigma-Aldrich, catalog # 5383410001) was prepared in DMSO at a stock concentration of 10 mM. Palbociclib (Selleck Chemicals, catalog # S1116) was prepared in DMSO at a stock concentration of 10 mM. GSK126 (Selleck Chemicals, catalog # S7061) was prepared in DMSO at a stock concentration of 10 mM. LBH589 (Selleck Chemicals, catalog # S1030) was prepared in DMSO at an initial concentration of 10 mM and diluted to 0.1 mM for a working stock solution. Decitabine (Selleck Chemicals, catalog # S1200) was prepared in DMSO at a stock concentration of 10 mM. Sorafenib (Selleck Chemicals, catalog # S1040) was prepared in DMSO at a stock concentration of 10 mM. UNC0642 (Selleck Chemicals, catalog # S7230) was prepared in ethanol (Decon labs) at a stock concentration of 10 mM.

### Plasmid purification and cloning

Bacterial cultures of pLenti-CMV-eGFP-PURO (Addgene, catalog # 17448) and pCR4-TOPO-GCGR (Dharmacon, catalog # MHS6278-202857850) were grown in 1X LB (Difco) plus Carbenicillin (Sigma-Aldrich, catalog # c1389) overnight at 30 °C degrees Celsius with shaking. DNA plasmid minipreps were performed according to the kit manufacturer (Qiagen, catalog # 27106). GCGR cDNA was PCR-amplified to include XbaI and SalI restriction enzyme sites with the forward primer (5’-GATACTTCTAGAATGCCCCCCTGCCAGCC-3’) and reverse primer (5’-GATACTGTCGACTCAGAAGGGGCTCTCAGCCA-3’), respectively. GCGR cDNA and pLent-CMV-eGFP plasmid were digested with XbaI and SalI, and purified following agarose gel electrophoresis with QIAquick gel extraction kit (catalog # 28706). Purified, digested GCGR cDNA and pLenti-CMV vector backbone were then ligated and used to transform TOP10 (OneShot) cells. Colonies with successful ligations were picked and re-streaked on LB-Carbenicillin plates overnight at 30 degrees Celsius. New minipreps were made on a few colonies and validated by Sanger sequencing with the CMV forward primer (CGCAAATGGGCGGTAGGCGTG) at the Children’s Hospital of Philadelphia Sequencing Core Facility.

### Lentiviral infection and siRNA transfection

To generate lentivirus for stable integration and expression of eGFP or GCGR, approximately 400,000 293T cells were seeded in 6-well plates without PenStrep. The following day, cells were transfected with 3 plasmids prepared in OPTI-MEM (Gibco, catalog # 31985070): 3 μg of pLenti-eGFP or pLenti-GCGR, 3μg of psPAX2, and 0.3 μg of pMDG.2 with 3 μl FuGENE reagent (Promega, catalog # E2691) per well. The next day, media was aspirated and fresh 10–30% FBS-containing media was added to each well for 24–48-h incubation. Virus was harvested by filtering media through a 0.45 μm filter (Millex-HV). Viral solutions were aliquoted into cryovials and stored in −80 °C and all supplies in contact with virus were bleached. Liver cancer cells were infected with 250–1000 μl of virus in 1.5 ml total media, containing 8 μg/ml polybrene (Sigma-Aldrich, catalog # 107689). After 24–48 h, stably expressing eGFP- or GCGR-cells were selected by puromycin (Sigma-Aldrich, catalog # P9620) at a concentration range between 1 and 5 μg/ml. For siRNA transfections, pooled siRNAs were purchased from Dharmacon to target CREB1 (catalog # L-003619-00-0005) and controls, Cyclophilin B (catalog # D-001820-10-20) or a non-targeting sequence (catalog # D-001810-10-05). Transfections were performed according to the manufacturer’s recommendation. SNU398 cells were seeded in 6-well plates at approximately 30–40% confluency (50,000–100,000 cells) and at the indicated time point were transfected with 25 nM of siRNA in OPTI-MEM with 2–6 μl lipofectamine (Fisher Scientific, catalog # 13778030) per well. The next day, media was aspirated and cells were given the appropriate, fresh media.

### Proliferation (cell # or density) assays

Cells were seeded in 6-well or 12-well plates at approximately 30–40% confluency (50,000–100,000 cells for 6-well and 25,000–50,000 cells for 12-well) in 10% FBS-containing RPMI. The following day, media was aspirated and the experimental conditions were added, with this process repeated over the duration of the experiment, as described in each figure. For cell number quantifications, at the indicated time points, media were aspirated, washed with DPBS, trypsinized with 0.5 ml (6-well) or 0.25 ml (12-well), and then neutralized with equivalent volumes of 10% FBS-containing media. Next, 10 μl of cells was mixed with 10 μl of 0.4% Trypan Blue (Gibco, catalog # 15250061) and finally counted in a Countess II (Life Technologies), with live cell/ml concentrations corrected for a 1:1 dilution. Quantifications were further analyzed in Microsoft Excel and Prism 9. For crystal violet colorimetric assays, at the indicated time points, media were aspirated and then 1 ml (6-well) or 0.5 ml (12-well) of 0.5% crystal violet (Sigma-Aldrich, catalog # C6158)/20% methanol (Sigma-Aldrich, catalog # 179337) solution in ddH_2_O was added down the sides of each well. Cells were incubated with crystal violet for 10 min with gentle rocking. Crystal violet solutions were aspirated and then cells were washed twice with DPBS. For the last wash, cells were gently rocked in DPBS for at least an hour to help remove background staining. Following DPBS aspiration, plates were inverted and dried overnight. Images were taken by a scanner (Epson Perfection 4490 Photo, discontinued) and assembled into figure format in Adobe Photoshop 2020. Quantification of crystal violet staining was performed by adding an equivalent volume of 99.8% methanol, incubating plates at room temperature for at least 1 h, and then reading absorbance at OD 570 nm [29].

### Viability (ATP-based) assays

Cells were seeded in white, opaque, flat bottom 96-well plates (Corning, catalog # CLS3917) at approximately 30–40% confluency (1000–4000 cells) in 50 μl of 10% FBS-containing RPMI. The following day (day 0), 50 μl of experimental media conditions at a 2× concentration were added to the appropriate wells and incubated for the indicated durations. At day 0, 50 μl of normal media was added to a separate plate for a baseline reading. For all readings, 50 μl of Cell Titer Glo reagent (Promega, catalog # G9242) was added directly to each well and incubated at room temperature while shaking for 10 min. Luminescence was measured by a microplate reader (SpectraMax M2, Molecular Devices) with the settings at white/opaque plate, top read, and 3 reads per sample rate. Further numerical analysis, such as normalization to baseline, were calculated with Microsoft Excel and Prism 9.

### Apoptosis (propidium iodide and annexin v-based) flow cytometry

Cells were seeded in 6-well plates at approximately 30–50% confluency (50,000–200,000 cells) in 10% FBS-containing RPMI. The following day, media was aspirated and the experimental conditions were added, with this process repeated over the duration of the experiment, as described in each figure. At the endpoint, media was collected in 15 ml conical tubes, cells were washed with DPBS, which was then collected in the same tubes, and then 0.5 ml of trypsin was added to each well. Upon detachment, cells were transferred into their respective tubes and pelleted by centrifugation at 2000 rpm for 5 min. Supernatants were discarded and rims of tubes dried by kimwipe. Cell pellets were resuspended in 110 μl of staining solution containing 5% Annexin V, 5% Propidium Iodide, and 90% 1X binding buffer (BD Bioscence, catalo # 556547). Next, cells were filtered through a 35-μm strainer cap in FACS-compatible tubes (MTC Bio, catalog # T9005) and incubated in the dark for 15 min at room temperature. Three hundred microliters of 1× binding buffer was added, vortexed, and then flow analysis was performed with a BD FACSCalibur. Stained cells were kept on ice and protected from light when not being processed. Gates were drawn to obtain data for at least 10,000 single cell events. FlowJo software was used to further process data.

### Gene expression analysis

For patient data, mRNA expression was obtained from The Cancer Genome Atlas (TCGA) (https://www.cbioportal.org). Raw RNA-seq reads were normalized and presented as log2 values by Dr. John Tobias (University of Pennsylvania). These calculations were graphed and statistically analyzed using Prism 9. Kaplan-Meier probability curves for overall survival comparing expression of a given gene was obtained from the website tool, https://kmplot.com [30]. Briefly, a TCGA RNA-seq dataset for liver cancer with 364 patient tumor samples was assessed for most statistically significant correlation between high vs. low gene expression and the probability of patient survival. For experimental studies in general, cells were seeded in 6-well plates in 10% FBS-containing RPMI at approximately 30–50% confluency (50,000 - 200,000 cells) for < 24 h durations or at 50–70% confluency (200,000–400,000 cells) for > 24 h time points. At the designated endpoint, media was aspirated, cells were washed with 1ml of 1X DPBS on ice, aspirated again, and then RNA extraction was performed with the Qiagen RNeasy kit (Qiagen, catalog # 74104), following the manufacturer’s protocol. RNA concentrations were determined using a NanoDrop 1000 (Thermo Fisher Scientific). Next, between 0.25 and 1 μg of RNA was reverse transcribed into cDNA with the High-Capacity RNA-to-cDNA kit (Applied Biosystems, catalog # 4388950). A ratio of 10 μl buffer and 1 μl enzyme per 20 μl total volume was used. Reactions were prepared in strip tubes (Thermo Fisher Scientific, catalog # AB-0773) and a RT-PCR cycle program was run in a C1000 Thermal Cycler (BioRad) with reaction settings of 37/36:00, 95/3:00. cDNA samples were diluted in ddH_2_O by 10-20-fold depending on the amount of RNA used. qPCR of target genes was performed in a ViiA7 using 5.4 μl of cDNA with 0.6 μl TaqMan primers (Thermo Fisher Scientific) per reaction and 6.6 μl (6 μl buffer, 0.6 μl enzyme) of TaqMan Fast Advanced Master mix (Life Technologies, catalog # 4444965). All qPCR reactions were performed in three technical triplicates. Raw Ct values were converted into ΔΔCt by first subtracting the technical replicate average of the housekeeping gene (RNA45S) from the Ct value of each target gene (=ΔCt). Then ΔCt values were converted to expression with the formula = 2^ − ΔCt. Expression values were then normalized to the triplicate average of the vehicle or primary human hepatocyte sample (=ΔΔCt). Taqman primers used for this work are the following: housekeeping gene *RNA45SR* (catalog # Hs03928985_g1), *GCGR* (catalog # Hs00164710_m1), *G6PC* (catalog # Hs02560787_s1), *FBP1* (Hs00983323_m1), *PCK1* (catalog # Hs00159918_m1).

### Protein analysis

For experimental studies in general, cells were seeded in 6-well plates in 10% FBS-containing RPMI at approximately 30–50% confluency (50,000–200,000 cells) for < 24 h durations or at 50–70% confluency (200,000–400,000 cells) for > 24 h time points. At the designated endpoint, media was aspirated, cells were washed with 1 ml of 1× DPBS on ice, aspirated again, and then 50–200 μl of whole cell extract lysis buffer (3% of 5 M NaCl, 1% of 1 M Tris pH 7.6, 0.1% SDS, 1% of 0.5M EDTA, and 1× protease/phosphatase inhibitor cocktail (Thermo Fisher Scientific, catalog # 78444) in ddH_2_O) was added to each well and incubated on ice for at least 10 min. Cells were scraped and transferred into a 1.5 ml microfuge tube. Following 10 s sonication pulses per tube, samples were spun down at 13,000 RPM for 10 min in 4 °C. Protein supernatants were pipetted into new 1.5 ml microfuge tubes. For subcellular fractionation, nuclear and cytosolic protein samples were extracted using the NE PER kit (Thermo Fisher Scientific, catalog # 78833), following the manufacturer’s protocol. Sample concentrations were performed by BCA (Pierce), following the manufacturer’s protocol, and calculated in Microsoft Excel using trendline analysis comparing the OD 562 nm of samples to a standard curve of BSA. For western blots, depending on the concentration, between 5 and 40 μg of protein (in 1× sample buffer, 1% beta-mercaptoethanol (Sigma-Aldrich, catalog # M3148)) were loaded into SDS-acrylamide PAGE gels (4% stacking, 10–12% running) and ran at 100–130 V for 60–90 min. Proteins were then transferred onto nitrocellulose membranes in a transfer apparatus at 0.1A overnight in 4 °C. Membranes were blocked with 5% nonfat milk in 1× TBST for approximately 1 h at room temperature. Following a few washes with 1X TBST, membranes were cut with scissors at the appropriate sizes and incubated with the corresponding primary antibodies (prepared in 1× TBST, 5% BSA, 0.01% sodium azide) overnight at 4 °C with rocking. The following day, antibody solutions were pipetted into original tubes for future use, membranes washed 2–3 times with 1× TBST, and then incubated with 1:10,000 secondary antibody conjugated to HRP for 1 h at room temperature with rocking. Antibody solutions were discarded and membranes were washed 3 times with 1× TBST over the course of an hour. Autoradiography film processing of membranes was performed in a dark room. Films were scanned and protein band images were cropped for figure production by Adobe Photoshop. Primary antibodies used for this work are the following: β-Actin (Cell Signaling Technology, catalog # 3700), GAPDH (Cell Signaling Technology, catalog # 2118), GCGR (Invitrogen, catalog # PA5-50668), eGFP (Thermo Fisher Scientific, catalog # CAB4211), p-PKA substrates (Cell Signaling Technology, catalog # 9624S), p-CREB S133 (Cell Signaling Technology, catalog # 9198), total CREB (Cell Signaling Technology, catalog # 9197), H3K27me3 (Cell Signaling Technology, catalog # 9733), H3K27Ac (Cell Signaling Technology, catalog # 8173), total H3 (Cell Signaling Technology, catalog # 14269), DNMT1 (Cell Signaling Technology, catalog # 5032), cleaved PARP (Cell Signaling Technology, catalog # 5625), cleaved Caspase-3 (Cell Signaling Technology, catalog # 9664), Cyclophilin B (Abcam, catalog # ab16045), and p-CaMKII T286 (Cell Signaling Technology, catalog # 12716).

### ELISA assays

For cAMP quantification, cells were seeded in 6-well plates in 10% FBS-containing RPMI at approximately 30–50% confluency (50,000–200,000 cells) for < 24 h durations or at 50–70% confluency (200,000–400,000 cells) for > 24 h time points. At the designated endpoint, media was aspirated, cells were washed with 1 ml of 1× DPBS on ice, aspirated again, and then 200 μl of 1 N HCl was added to each well for lysis. The remaining steps were carried out according to the manufacturer’s protocol (Enzo Life Sciences, catalog # ADI-900-066). For serum glucagon quantification, blood was initially collected by retroorbital draw and allowed to clot at room temperature for one hour. Samples were then centrifuged at 3000 RPM for 10 min in 4 °C and serum supernatant was transferred into a new 1.5 ml microfuge tube. From here, quantification of glucagon was determined by following the manufacturer’s protocol (R&D Systems, catalog # DGCG0). Sigmoidal regression analysis and extrapolations were calculated in Prism for final concentrations.

### Mouse experiments

All experiments described were approved by IACUC at the University of Pennsylvania. All mice used for xenograft experiments were 4–6 weeks old Nu/J females (Jackson Laboratory, catalog # 002019). Following a few days of acclimation, mice were anesthetized with isofluorane and 1–2 million SNU398 liver cancer cells (in a 1:1 mixture of DPBS:matrigel) were subcutaneously injected into both flanks. After approximately 2–3 weeks, xenograft tumors reached an average of 100 mm^3^, caliper measured, at which point experimental treatments began. The EZH2 inhibitor, GSK126, and pan-HDAC inhibitor, LBH589, were prepared at the indicated concentration in 20% 2-hydroxypropyl-β-cyclodextrin (Cayman Chemical, catalog # 16169) pH 4.5 either as a single agent or in combination. Two hundred microliters of the drug solutions was intraperitoneally injected once daily unless mice displayed toxicity symptoms, such as severe weight loss and lethargy, at which point drugs were administered irregularly following weight recovery. Once tumors reached 2000 mm^3^, mice were euthanized by CO_2_, followed by cervical dislocation. Tumors were resected and frozen on dry ice for further processing. Tumor volumes were calculated by the following equation: (π/6) × (width^2^) × (length), where width is always the shorter parameter.

## Results

### Gluconeogenic proteins are downregulated in glucose-dependent liver cancer models

**Fig. 1:**
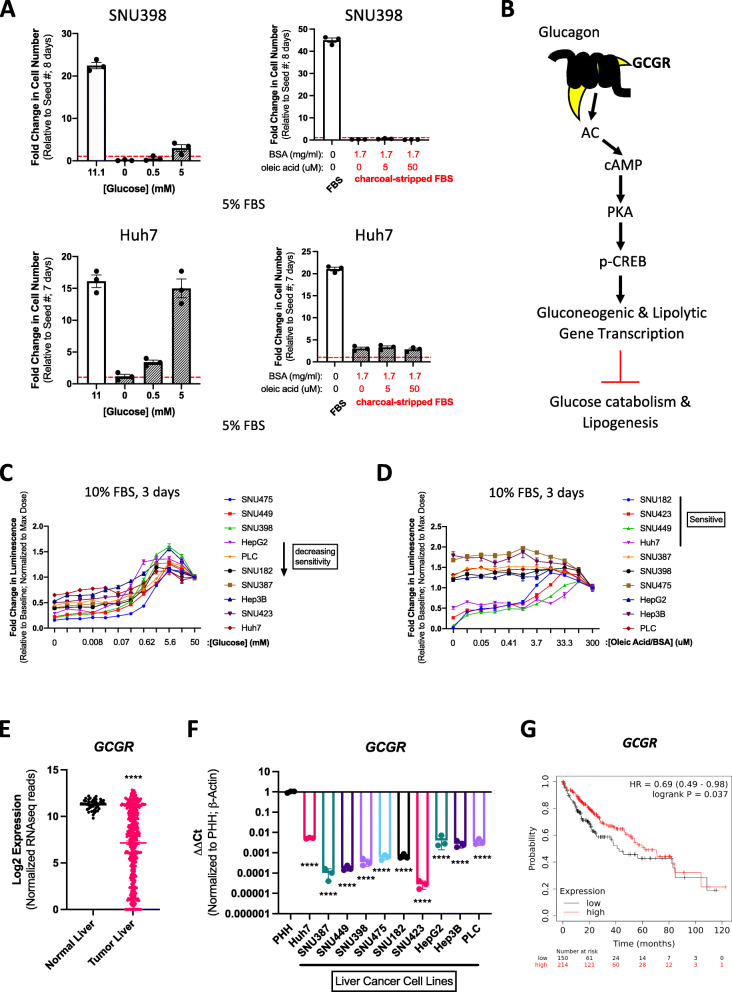
HCC cell lines are dependent on exogenous glucose/lipid dependency and express low levels of the glucagon receptor, GCGR. **a** Cell proliferation assays of two cell lines, SNU398 and Huh7, at the indicated time point and nutrient conditions. Data represents a single experiment with 3 biological replicates. Red dashed line denotes fold change of 1, which refers to the starting number of cells. **b** Simplified Glucagon/GCGR signaling transduction. GCGR: g-coupled glucagon receptor, Ac: adenyly cyclase, cAMP: cyclic adenosine monophosphate, PKA: protein kinase A, p-CREB: phosphorylated cAMP-response element binding protein. **c** ATP-based cell viability assay of HCC cell lines cultured across a wide range of glucose concentrations. Data points represent the average of 6 biological replicates. **d** ATP-based cell viability assay of HCC cell lines cultured across a wide range of oleic acid concentrations. Data points represent the average of 6 biological replicates. **e** Normalized RNA-seq values for *GCGR* in human HCC compared to normal liver. Data obtained from TCGA. ****: *p* < 0.0001, unpaired two-tailed *t* test. *n* = 50(normal) and 374(tumor). **f** qPCR mRNA expression of *GCGR* in HCC cell lines compared to Primary Human Hepatocytes (PHH). Data represent a single experiment with 3 biological replicates (3 separate RNA samples). ****p < 0.0001, ordinary one-way ANOVA with Dunnett’s multiple comparisons test. **g** Kaplan-Meier plot of overall survival probability between high and low *GCGR* expression in liver cancer patients. Graph was generated using the website: https://kmplot.com

To provide a rationale for targeting glucose metabolism in liver cancer via glucagon-stimulated gluconeogenesis, we probed the necessity of glucose for cell viability. Cells that require more glucose may exhibit increased vulnerability to agents stimulating gluconeogenesis, such as glucagon. To that end, 11 established cell line models of liver cancer (i.e., hepatocellular carcinoma [HCC], hepatoblastoma, and hepatoma) with various disease etiologies and oncogenic driver mutations (see “Methods” section), were cultured *in vitro* under glucose-limiting conditions and cell numbers quantified after approximately one week of growth. All cell lines tested were unable to proliferate in 0 mM glucose conditions, and in particular, SNU398, SNU182, and SNU475 HCC cells failed to fully recover even in physiological 5 mM glucose concentrations (Fig. 1A, and Supplementary Figure 1A).

Previous studies demonstrated a key role of hepatic lipolysis, via the activation of inositol triphosphate receptor 1 and adipose triglyceride lipase, in glucagon-stimulated gluconeogenesis [31]. Moreover, the metabolic effects of glucagon contribute to hepatic fat clearance in fatty liver disease models [32], suggesting glucagon-induced depletion of lipid stores may reduce viability in liver cancer cells that demand more fatty acids for growth. To examine lipid dependency, we compared endpoint proliferation between cells cultured with normal serum, delipidated serum, or delipidated serum with oleic acid supplementation. Unlike glucose, more varied results were obtained across cell lines (Fig. 1A, and Supplementary Figure 1B), where SNU387, SNU449, and SNU475 cells were largely resistant to lipid deprivation. This lipid phenotypic distribution did not appear to be due to relative growth rate or specific oncogenic driver (Supplementary Figure 1C, D), which may simply indicate that not all cell lines are able to perform adequate *de novo* lipogenesis under nutrient deprivation. Short-term cell viability assessment of growth recapitulated these findings across all cell lines tested, reinforcing the conclusion that all liver cancer cells require glucose but not lipids (Fig. 1C, D). At the very least, this highlights a critical role of glucose in the growth of established liver cancer cells, which is not ubiquitously observed in all cancers, like soft tissue sarcomas [33].

The inability of liver cancer cells to proliferate under 0mM glucose supports the hypothesis that any process that antagonizes glucose utilization, such as gluconeogenesis, may suppress tumor growth. Gluconeogenesis is the direct biochemical reversal of glycolysis, whereby three rate-limiting enzymes, PCK1, FBP1, and G6PC, collectively synthesize glucose and ultimately release it from hepatocytes (Supplementary Figure 1E). In both liver cancer cell lines and patients, all major gluconeogenic genes are significantly downregulated in tumors compared to primary human hepatocytes (PHH) and normal liver tissue (Supplementary Figure 1F, G). In contrast, this expression pattern was not observed across all glycolytic genes (Supplementary Figure 2A), suggesting that gluconeogenic dampening may be more vital than direct glycolytic acceleration in liver cancer development.

We hypothesized that silencing across all gluconeogenic genes involved a deficient upstream node of the pathway. Physiologically, hepatocytes initiate production of glucose upon prolonged glucagon signaling via its receptor, GCGR, which stimulates a cascade of signaling events mediated by cAMP and PKA that activate transcription factors, such as CREB, to induce gluconeogenic and lipolytic gene expression (see Fig. 1B). Similar to the gluconeogenic enzymes, *GCGR* is also significantly downregulated at the mRNA level in both liver cancer patient samples and cell lines (Fig. 1E, F). Whereas normally, GCGR is most abundant in the liver versus any other tissue type, we also detected a comparable decrease in *GCGR* expression in the immortalized, “normal” hepatocyte cell line, THLE-3 (Supplementary Figure 2B). These data suggest that either artefactual contributions from 2D growth or early mutagenic events inhibiting tumor suppressor genes may account for *GCGR* repression in liver cancer. Regardless, based on RNA-seq data analyzed by the website tool, https://kmplot.com (see “Methods” section), tumors with lower gene expression of *GCGR* and gluconeogenic enzymes correlate with shorter overall survival in liver cancer patients (Fig. 1G and see Supplementary Figure 1H, respectively).

### Overexpression of GCGR activates glucagon-mediated signaling transduction via cAMP in SNU398 cells

**Fig. 2:**
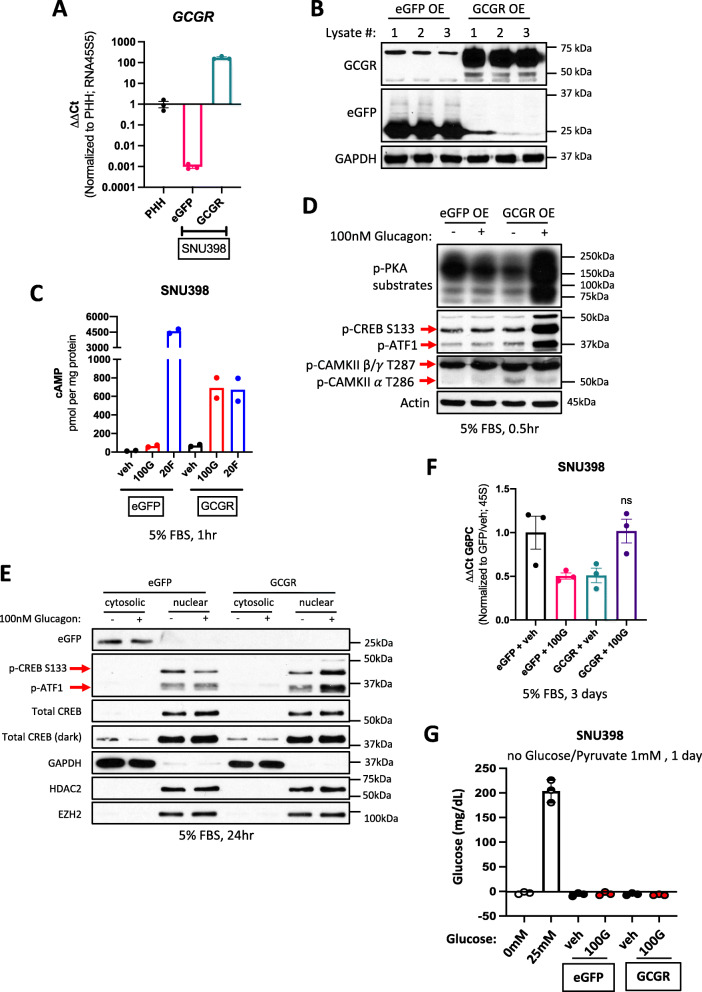
GCGR overexpression partially sensitizes SNU398 to glucagon signaling. (a) qPCR mRNA levels of *GCGR* in SNU398, following lentiviral CMV-driven mammalian expression, compared to Primary Human Hepatocytes (PHH). Data represent a single experiment with 3 biological replicates (3 separate RNA samples). eGFP used as a control. **b** Protein assessment of GCGR and eGFP overexpression in SNU398. Lysate number denotes independent protein sample. **c** Quantification of cAMP in SNU398 expressing either eGFP or GCGR and treated with 100 nM glucagon (100 G). Data points represent a single experiment of 2 technical replicates. veh: vehicle (0.05 M acetic acid), 20F: 20uM forskolin (positive control). **d** Protein analysis of downstream effectors of cAMP signaling in SNU398 cells expressing either eGFP or GCGR and treated with 100 nM glucagon (+). (−): vehicle treated. **e** Protein localization of p-CREB following glucagon treatment of SNU398 cells expressing either eGFP or GCGR. **f** qPCR mRNA expression of *G6PC* in SNU398 cells expressing either eGFP or GCGR and treated with 100nM glucagon (100 G). Data represent a single experiment with 3 biological replicates (3 separate RNA samples). Error bars: ±SEM. veh: vehicle (0.05M acetic acid). ns: not significant, *p* > 0.05, ordinary one-way ANOVA with Tukey’s multiple comparisons test. **g** Glucose quantification in SNU398 expressing either eGFP or GCGR and cultured in no glucose media with 1 mM pyruvate for 1 day. 0mM and 25mM glucose media was used as control

Based on strict glucose requirements for cell growth, a ubiquitous decrease in *GCGR* mRNA in liver cancer samples, and the positive correlation between *GCGR* expression and patient survival, we hypothesized that glucagon signaling downstream of GCGR may restore gluconeogenic expression and drive anti-tumorigenic effects. To examine this possibility, *GCGR* cDNA was overexpressed in SNU398 HCC cells, which notably harbor a constitutively active mutation in β-catenin. RNA and protein analysis verified the supraphysiologic overexpression of GCGR in SNU398 cells (Fig. 2A, B). To determine whether ectopic GCGR overexpression effectively promotes adenylyl cyclase activity in response to glucagon, control (“eGFP-expressing”) or GCGR-overexpressing cells (“SNU398 GCGR”) were treated with glucagon and assayed for cAMP. SNU398 GCGR cells reproducibly generated cAMP in response to glucagon exposure, which in some cases was as high as the positive control condition, forksolin, an agonist of adenylyl cyclase (Fig. 2C, Supplementary Figure 2C), thus confirming that glucagon/GCGR signaling was functional in this system.

To further investigate glucagon signaling, cAMP-dependent PKA activity was assessed by phosphorylation of PKA-substrates. SNU398 GCGR cells stimulated with glucagon showed rapid increases in phosphorylation of PKA targets, including p-CREB S133 (Fig. 2D). Because G-coupled protein receptors can also activate phospholipase C and the subsequent release of calcium ions from the endoplasmic reticulum, we probed for a common effector of glucagon and Ca2+ signaling, CAMKII, but did not observe any difference in any of its activated isoforms (Fig. 2D). However, we further validated the phosphorylation of CREB by GCGR stimulation over a longer period of time and range of glucagon concentrations. Phosphorylation of CREB at S133 was maximal with 100 nM glucagon, specifically in SNU398 GCGR cells, with no change in total CREB (Supplementary Figure 2D). Although there are other post-translational modifications reported for CREB, the phospho-S133 site is thought to enhance the recruitment of necessary transcriptional co-activators [34]. Since previous studies in cancer models demonstrated instances of mitochondrial CREB localization [35], we performed nuclear fractionations on SNU398 eGFP or GCGR cells treated with glucagon to verify if activated p-CREB S133 was spatially capable of facilitating gluconeogenic gene transcription. A glucagon/GCGR-dependent increase in p-CREB was detected in nuclear fractions (Fig. 2E), suggesting that the canonical cAMP-PKA-CREB pathway downstream of glucagon signaling was functionally intact in SNU398 GCGR cells.

With increased nuclear CREB, we next quantified the relative mRNA abundance of *G6PC*, *FBP1*, and *PCK1*, the transcriptional output of glucagon-mediated gluconeogenesis. Upon glucagon treatment of SNU398 GCGR cells for either 3 or 5 days, *G6PC* mRNA levels did not significantly increase relative to control cells (Fig. 2F, Supplementary Figure 2E). Moreover, both *FBP1* and *PCK1* mRNA expression was undetected, regardless of condition (data not shown). We were also unable to detect *bona fide* production of glucose from exogenous pyruvate in SNU398 GCGR cells (Fig. 2G). These data imply that glucagon signaling in SNU398 GCGR cells, was unable to transmit completely to gluconeogenic gene transcription. Other cell line models employing this GCGR overexpression strategy were similarly examined, and whereas we did measure comparable *GCGR* expression (Supplementary Figure 2F) and elevated cAMP concentrations upon glucagon treatment (Supplementary Figure 2G), PKA activity and p-CREB were not discernably increased compared to SNU398 (Supplementary Figure 2H). Moreover, no substantial changes in gluconeogenic gene expression were measured either (Supplementary Figure 3A).

In contrast to SNU398 GCGR cells, primary human hepatocytes (PHH) treated with glucagon displayed trends (1.5–3-fold) towards an increase in gluconeogenic gene expression for all 3 enzymes, alongside a drop in *GCGR* mRNA abundance (Supplementary Figure 2I). However, it is unclear if this level of change accurately reflects the physiological response to glucagon in humans, as studies in zebrafish, rat, and murine hepatocytes have measured increases in *PCK1* mRNA anywhere from 4-to-20-to-500-fold upon glucagon treatment, respectively [36–38]. Of note, we did not observe a decrease in *GCGR* expression with glucagon treatment in SNU398 or other liver cancer cell lines, but rather a consistent increase (Supplementary Figure 2J). We hypothesize this could be due to increased levels of the transcription factor, carbohydrate-responsive element-binding protein (ChREBP), in liver cancer [39] that has been shown to positively regulate *GCGR* expression in rat hepatocytes [40]. Collectively, our data indicate that glucagon cannot induce a uniform augmentation in gluconeogenic gene expression in liver cancer cell lines and that SNU398 cells are most responsive to glucagon upon GCGR overexpression, in terms of downstream signaling.

### Co-treatment of SNU398 GCGR cells with glucagon and epigenetic inhibitors cannot fully restore gluconeogenesis

**Fig. 3:**
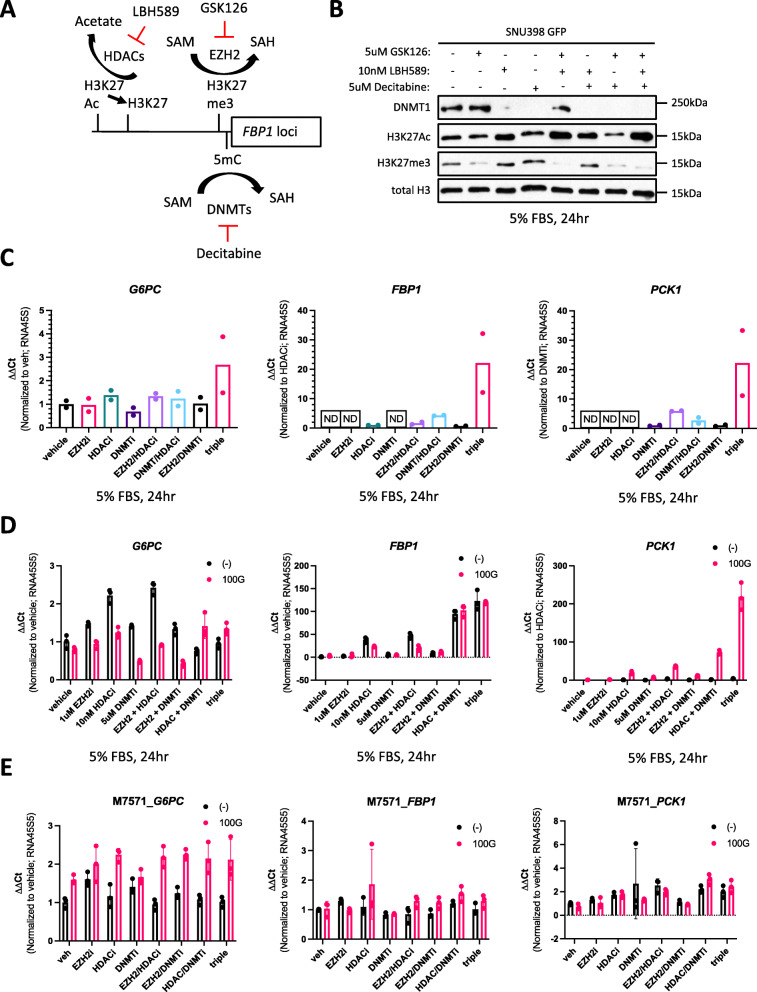
Epigenetic inhibitors fail to fully restore gluconeogenic gene expression with or without glucagon. **a** Diagram of epigenetic drugs and their targets. GSK126: EZH2 (enhancer of zeste homolog 2) inhibitor, SAM: S-adenosyl-L-methionine, SAH: S-adenosyl homocysteine, H3K27me3: histone 3 lysine 27 trimethylated, LBH589: pan-HDAC (histone deacetylase) inhibitor, H3K27Ac: histone 3 lysine 27 acetylated, Decitabine: DNA methylation (DNMT -DNA methyltransferase) inhibitor, 5mC: 5-methylcytosine. **b** Protein analysis of epigenetic inhibitor efficacy in SNU398 eGFP-expressing cells at the indicated drug concentrations, culture conditions, and time. **c** qPCR mRNA levels of gluconeogenic genes in SNU398 expressing eGFP and treated with epigenetic inhibitors at the concentrations used in **b**. Data represent a single experiment with 2 biological replicates (2 separate RNA samples). ND: not detected. **d** qPCR mRNA levels of *G6PC*, *FBP1*, and *PCK1* in SNU398 GCGR-overexpressing cells treated with glucagon plus combinations of epigenetic drugs. Data represent a single experiment with 3 technical replicates (1 RNA sample). Error bars: ±SD. (−): no drug, 100 G: 100 nM glucagon, vehicle: 0.035% of 0.05 M acetic acid, EZH2i: 1 μM GSK126, HDACi: 10 nM LBH589, DNMTi: 5 μM Decitabine, triple: 1 uM GSK126 + 10 nM LBH589 + 5 uM Decitabine. **e** Same qPCR mRNA analysis as in **d** but using the liver cancer patient-derived cell line, M7571

Our data show a signaling cascade from extracellular glucagon to nuclear CREB in SNU398 GCGR cells, yet this fails to effectively induce gluconeogenic gene transcription, which we hypothesized would antagonize glycolysis to reduce tumor growth. One approach to promote transcriptional activation is through inhibiting heterochromatic epigenetic modifications. Previous studies on *FBP1* loci indicated that biochemical alterations to histones and DNA functionally correlate with heterochromatin formation and gene silencing. Specifically, promoter-rich methylated cytosine residues [41], non-acetylated histone 3 lysine 27 in enhancer regions [42], and chromatin interaction of the histone methyltransferase, EZH2 [43], have all been identified as mechanisms of epigenetic repression for *FBP1* in liver cancer. Therefore, we hypothesized that glucagon/GCGR signaling requires chromatin accessibility in order to fully activate gluconeogenic gene expression and accompanying metabolic programs. To that end, we tested the efficacy of 3 epigenetic inhibitors targeting either EZH2-specific histone methylation (GSK126), HDAC-mediated histone deacetylation (LBH589), or DNMT-catalyzed cytosine methylation (Decitabine) (Fig. 3A). For GSK126, decreased EZH2 catalytic activity reduces lysine 27 trimethylation in histone 3 (H3K27me3) [44]. For LBH589, pan-HDAC inhibition results in broad increases in histone acetylation [45]. And lastly, for Decitabine, a reduction in DNA methylation is at least partially through DNMT degradation [46]. As determined by western blots, all 3 compounds were effective in SNU398 (Fig. 3B). To further confirm their efficacy, we treated SNU398 and other cell lines at ranges of drug concentrations for different durations and found similar effectiveness on their respective target enzymes (Supplementary Figure 3B–E).

Since previous studies assessing gluconeogenic gene expression following treatment with these epigenetic drugs were performed as single agents in liver cancer models, we first tested whether combinations of epigenetic inhibitors themselves could restore gluconeogenic gene expression to any meaningful degree. After 24 h of drug treatment, we measured approximately 20-fold increases in expression of *FBP1* and *PCK1* with the triple combination in SNU398, while *G6PC* mRNA level was largely unchanged across all drug permutations (Fig. 3C). Next, we tested whether GCGR-overexpressing SNU398 cells stimulated with glucagon would be more amenable to gluconeogenic gene expression with the epigenetic inhibitors. Interestingly, we observed a different effect on mRNA levels for each gluconeogenic enzyme: (1) *G6PC* expression, again, remained unchanged regardless of glucagon/GCGR signaling or epigenetic inhibition, (2) *FBP1* abundance was more dependent on epigenetic regulation than glucagon/GCGR stimulation, and (3) *PCK1* mRNA levels were substantially elevated only when both glucagon/GCGR signaling and epigenetic inhibition were present (Fig. 3D). Importantly, the relative 200-fold increase in *PCK1* expression with the triple combination plus glucagon/GCGR is still at least an order of magnitude less than the *PCK1* levels in normal hepatocytes (see Fig. 1F).

We next examined a recent patient-derived xenograft cell line, M7571, to determine whether a more patient-proximal liver cancer model was amenable to restoration of gluconeogenic gene expression after treatment with glucagon and our panel of epigenetic inhibitors. We quantified a maximal 2-fold increase in gene expression of the gluconeogenic enzymes under any condition tested in M7571 cells (Fig. 3E). However, contrary to the established SNU398 cell line, M7571 cells (derived from “PDX tissue #1”) have 100X mRNA quantities of *GCGR*, and are more comparable to primary human hepatocytes (Supplementary Figure 4A). That being the case, M7571 cells still display reduced *GCGR* expression which may explain why neither the epigenetic inhibitors nor glucagon treatment substantially increased GCGR or gluconeogenic gene expression (Fig. 3E, Supplementary Figure 4B). We hypothesize that little epigenetic repression at gluconeogenic gene loci coupled with low *GCGR* expression makes M7571 cells less responsive to epigenetic inhibitors and glucagon, respectively. Finally, the effects of epigenetic inhibitors on gluconeogenic gene expression were tested on multiple liver cancer cell lines with little effects on any of them (Supplementary Figure 4C). Combinations of EZH2 and HDAC inhibitors affected the growth of some liver cancer cells, but did not correspond to gluconeogenic gene expression (Supplementary Figure 4D; see Supplementary Figure 1F).

Although the epigenetic inhibitors failed to fully restore gluconeogenic gene expression in SNU398, cell growth was noticeably affected at specific drug concentrations and combinations. To provide a systematic analysis of this effect, the viability of multiple liver cancer cell lines was measured by relative ATP abundance across pharmacologically relevant doses of each epigenetic drug (Supplementary Figure 5A). We included the FDA-approved receptor tyrosine kinase inhibitor, Sorafenib, as a clinically meaningful comparison, as well as another experimental inhibitor, UNC0642, targeting the histone methyltransferase, G9a, which has garnered recent appreciation for its contributions to cancer progression. Our data reveal a broad scope of responses across liver cancer cell lines. In general, cell lines like SNU449 exhibited increased tolerance to epigenetic inhibitor treatment, whereas other cell lines like SNU398 displayed greater sensitivity. Interestingly, SNU398 cells showed severe growth reduction with the dual treatment of the EZH2 inhibitor (GSK126) and the pan-HDAC inhibitor (LBH589), suggesting a potential therapeutic window for the drug combination (Supplementary Figure 5B).

To further characterize the *in vivo* effects of EZH2/HDAC inhibition on SNU398 cells, we generated xenograft tumors in Nu/J mice. Once tumor volume reached an average of 100–200 cm^3^, mice were treated intraperitoneally with the epigenetic drugs at doses previously published by other groups. Although treatment with anti-EZH2/HDAC compounds suppressed the growth of SNU398 xenografts, significant toxicity was prevalent, as measured by greater than 20% decreases in mouse body weight (Supplementary Figure 5C, D).

Overall, our data indicate that while various epigenetic inhibitors may be effective in attenuating heterochromatin repression of specific gluconeogenic gene loci under certain conditions in liver cancer cells, we conclude that epigenetic inhibition is not sufficient for glucagon-stimulated gluconeogenesis. Furthermore, although the epigenetic drugs present potential therapeutic avenues from viability data in multiple cell lines *in vitro*, it is unclear if this can be translated into clinical strategies or if patients would experience better quality of life over currently deployed targeted therapies, such as Sorafenib.

### SNU398 GCGR cells display reduced viability upon glucagon treatment through CREB independent mechanisms

**Fig. 4:**
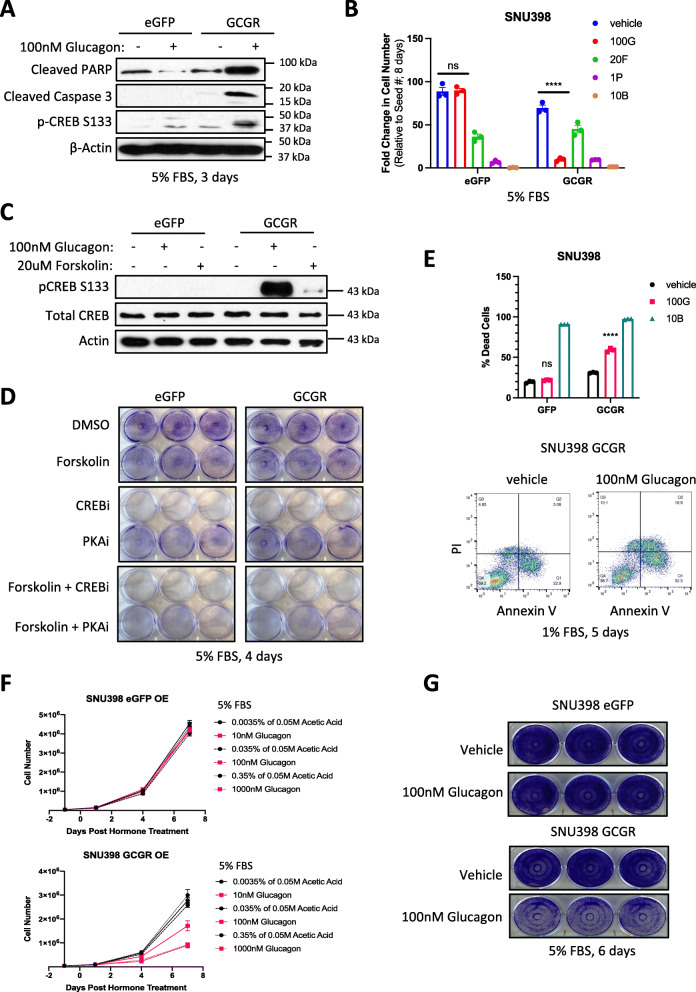
Glucagon treatment reduces in vitro cell viability of GCGR-overexpressing SNU398. **a** Protein assessment of apoptotic markers in SNU398 cells either expressing eGFP or GCGR and treated with 100 nM glucagon. **b** Cell proliferation assay of SNU398 cells either expressing eGFP or GCGR and treated with 100 nM glucagon (100G). Vehicle, 100 G, and 20F were added daily in fresh media. Data represent a single experiment of 3 biological replicates. Error bars: ±SEM. ns: not significant, ****p < 0.0001, two-way ANOVA with Tukey’s multiple comparisons test. 20F: 20 uM forskolin, 1P: 1 μM palbociclib (cell cycle inhibitor), 10B: 10 μg/ml blasticidin (cell death inducer). **c** Protein assessment of pCREB in SNU398 cells either expressing eGFP or GCGR and treated with 100 nM glucagon or 20 μM Forskolin. **d** Crystal violet assay on SNU398 cells either expressing eGFP or GCGR and treated with 20 μM Forksolin and/or 1 μM CREB inhibitor, and/or 5uM PKA inhibitor. **e** (Upper panel) Histogram of flow cytometry analysis of gated, single cells staining positive for Annexin V/Propidium iodide in SNU398 cells either expressing eGFP or GCGR and treated with 100 nM glucagon (100 G). Data represent a single, independent experiment of 3 biological replicates. Error bars: ±SEM. ns: not significant, ****: *p* < 0.0001, two-way ANOVA with Sidak’s multiple comparisons test. 10Blast: 10 μg/ml blasticidin (positive control). (Lower panel) Representative PI/Annexin V scatter plots for SNU398 GCGR with or without glucagon treatment. **f** Cell proliferation assay on SNU398 cells either expressing eGFP or GCGR and treated with glucagon. Data represent a single experiment with 3 biological replicates. Error bars: ± SEM. **g** Crystal violet assay on SNU398 cells either expressing eGFP or GCGR and treated with 100 nM glucagon (100 G)

Similar to the epigenetic inhibitors, while glucagon/GCGR stimulation was unable to restore gluconeogenic gene expression to physiological levels, we did observe a reproducible, apoptotic phenotype in SNU398 GCGR cells when treated with glucagon. The apoptotic protein markers, cleaved PARP and cleaved Caspase-3, were both induced specifically in SNU398 cells overexpressing GCGR upon glucagon exposure (Fig. 4A). Additionally, overall cell number was significantly decreased in SNU398 GCGR cells with persistent glucagon treatment (Fig. 4B). This reduction in cell growth was comparable to treatment with the cell cycle inhibitor, Palbociclib (1P), and also more pronounced than daily forskolin treatment, suggesting glucagon/GCGR may be either more efficient in signal transduction via cAMP or engage other pathways aside from cAMP signaling to enact tumor suppressive properties. For example, glucagon treatment of SNU398 GCGR cells resulted in significantly higher levels of pCREB S133 than forskolin treatment (Fig. 4C). However, this growth inhibition is independent of PKA and CREB activation, which would be expected to restore cell viability of cells treated with PKA and CREB inhibitors (Fig. 4D). Further validation of apoptosis engagement in these conditions was supported by increases in PI/Annexin V-positivity (Fig. 4E). According to previous studies, glucagon ligand binding to GCGR approaches saturation at the mid nanomolar range [47]. Indeed, we observed a dose-dependent decrease in cell number with increasing glucagon concentration that plateaued at 100 nM and began displaying anti-proliferative effects around 3–4 days in SNU398 GCGR cells and not SNU398 eGFP cells (Fig. 4F). Because serum concentration can have an impact on drug efficacy *in vitro*, upon continued examination of this glucagon/GCGR phenotype, we observed an optimal difference in growth at 5% serum, a lack of phenotype at 10% serum, and a highly unconducive condition for cell growth at 1% serum (Fig. 4G, data not shown). We tested this phenotype in numerous other liver cancer cell lines for glucagon/GCGR robustness but did not measure equivalent changes in cell number compared to SNU398 at 100 nM glucagon (Supplementary Figure 6A). These data suggest that SNU398 possesses a unique vulnerability to glucagon signaling, which may represent a subset of patients.

We have previously shown that SNU398 is the only cell line tested that shows an increase in p-CREB S133 with glucagon/GCGR (see Supplementary Figure 2H). Therefore, we surmised that CREB may be critical for a gluconeogenic-independent transcriptional program inducing cell death, specifically in SNU398. However, siRNA knockdown of CREB protein did not rescue SNU398 GCGR cell proliferation with glucagon treatment (Supplementary Figure 6B), despite reasonable reductions in levels of active p-CREB S133 (Supplementary Figure 6C). Even though we did not observe a substantial effect on viability from siCREB, we transitioned to a pharmacological approach with the CREB inhibitor, 666-15, which has been shown to disrupt binding critical for transcriptional activity [48] and reduce phosphorylation in AML models [49]. However, no decrease in p-CREB S133 with drug treatment was observed (Supplementary Figure 6D), and likewise, no rescue of cell death under the GCGR/glucagon/666-15 condition was measured (Supplementary Figure 6E).

## Discussion

Our study indicates that the HCC cell line SNU398 can be partially re-sensitized to glucagon, in terms of downstream signaling and biological effect, upon supraphysiologic levels of ectopic GCGR. This re-sensitization was unique to this specific cell line as others did not show a similar phenotype. Concordantly, we hypothesize that liver cancer cells themselves would be largely unaffected by circulating glucagon directly. Although, it remains to be seen if normal hepatocytes stimulated to synthesize glucose by glucagon secretion could provide local glucose, as tumor cells are thought to regulate metabolism of others cells within the microenvironment to suit their nutritional needs [50]. In this manner, gluconeogenesis could actually be oncogenic at the systemic level through nutrient partitioning and/or competition between normal and tumor cells. Diabetes is a risk factor for liver cancer development and is commonly characterized by abnormally high glucagon signaling, likely as a result of decreased insulin sensitivity [51]. In diabetic patients with liver cancer, we believe it important to study the potential cell non-autonomous effects of normal, glucagon-responsive hepatocytes as to their ability to facilitate tumor cell growth by releasing glucose into the tumor microenvironment.

In terms of inducing gluconeogenesis within tumor cells themselves, our data suggest that this may be an unlikely therapeutic approach for many patients with liver cancer. The mRNA expression of *GCGR*, *G6PC*, *FBP1*, and *PCK1* are decreased in patient tumors and heavily silenced in many established liver cancer cell lines. For these cell lines, neither epigenetic agents nor stimulation of glucagon signaling was sufficient to restore physiological gluconeogenic gene expression (which we infer does not reduce glycolytic flux), suggesting multiple and/or redundant mechanisms of transcriptional repression. In addition, modulation of protein stability and enzyme activity should also be considered. Taken together, this regulatory complexity may account for why cell lines are still unable to fully engage the entire gluconeogenic pathway despite enforced glucagon/GCGR signaling and inferred improvement of chromatin accessibility.

However, this is not to say that individual gluconeogenic enzymes could not have distinct roles under certain metabolic circumstances. For example, PCK1 expression and activity could be induced to funnel extra anaplerotic intermediates from the TCA cycle into serine/glycine biosynthesis to support one-carbon metabolism. FBP1-catalyzed production of fructose-6-phosphate can enter the pentose phosphate pathway to support nucleotide, lipid, and antioxidant synthesis. However, it is not clear how G6PC-regulated loss of intracellular glucose could promote survival under any situation. Interestingly, *G6PC* was the only gene not increased upon any combination of glucagon/GCGR or epigenetic inhibition, suggesting that the de-phosphorylation of glucose-6-phosphate, and its subsequent loss or lack of utilization, has no advantage for cell viability under cellular stress.

Three unresolved questions pertain to (1) the lack of gluconeogenic gene expression in glucagon-treated SNU398 GCGR cells, (2) mechanistic nature of the GCGR/glucagon phenotype in SNU398, and (3) this discrepancy between other liver cancer cell lines tested. While p-CREB S133 is important for stimulating *G6PC* and *PCK1* expression through recruitment of transcriptional coactivators such as CBP [52], there are other transcription factors, aside from epigenetic modifiers, that can also directly regulate gluconeogenic gene expression. Chromatin interactions between FOXO1 and PGC1α [53], HNF4a and PGC1α [54], as well as nuclear glucocorticoid receptors with RXRs [55], can also localize at gluconeogenic gene promoters to positively control expression. A successful gluconeogenic response in normal hepatocytes may require inputs from all of these factors, whereas insufficient components of these transcriptional machineries may embody cancer cells. This may explain why glucagon signaling alone is not enough in SNU398 GCGR cells. Mechanistically, CREB inhibition was insufficient to rescue the growth phenotype of glucagon-stimulated SNU398 GCGR cells. This may imply that another factor downstream of PKA, or multiple factors including CREB, are responsible. Utilizing pharmacological approaches, we attempted to target PKA, which has many other targets besides CREB, and Ca^+2^-activated CAMKII, which has also been shown to be an important effector of glucagon signaling. However, neither inhibition of PKA nor CAMKII resulted in an increase in SNU398 GCGR cell proliferation upon glucagon treatment. Because glucagon signaling has widespread pleotropic effects on cell metabolism, including glycogenolysis and lipid processing, it may be necessary to unbiasedly address metabolic rewiring before performing precise rescue experiments. Therefore, future studies on glucagon signaling in liver cancer could analyze metabolomics profiling of SNU398 GCGR cells with or without glucagon, compared to a cell line that is unresponsive, regardless of glucagon treatment. Differential analysis of metabolites may reveal the likely cause of sensitivity in SNU398 cells, answering a potentially impactful question of how glucagon signaling could disrupt cell viability independent of gluconeogenesis.

## Conclusion

This study reports 5 major findings: (1) liver cancer cells are robustly dependent on exogenous glucose for growth; (2) the glucagon receptor, GCGR, is downregulated at the mRNA level in both patient liver tumors and cell line models; (3) supraphysiologic levels of GCGR can re-sensitize SNU398 cells to glucagon treatment by enhanced cAMP production, PKA activity, and nuclear CREB phosphorylation; (4) neither glucagon/GCGR or epigenetic inhibitors are enough to completely restore gluconeogenic gene expression to that of primary human hepatocytes; and (5) SNU398 cells over-expressing GCGR uniquely, and reproducibly, display reduced viability upon glucagon treatment that appears to be independent of CREB. Overall, while the SNU398 glucagon/GCGR mechanism remains an outstanding question, due to the phenotype being observed from unrealistic levels of GCGR, a lack of signaling and growth effect robustness between cell lines, and the failure to fully restore gluconeogenic gene expression with glucagon and epigenetic inhibition, we conclude that glucagon and GCGR are not critical players in liver cancer biology. It seems possible that a subset of patients with sufficient GCGR and gluconeogenic enzyme accumulation might be candidates for this strategy, but most liver cancer patients would not. Moreover, we recommend that future research into gluconeogenesis and liver cancer focus on diabetic murine models of liver cancer, and whether there exists any clinically beneficial information between the relationship of insulin-resistant normal hepatocytes and glucagon-resistant transformed hepatocytes.

## Supplementary Information

**Additional file 1: Supplementary Figure 1.** Liver cancer cells display hypersensitivity to long- and short-term glucose and lipid withdrawal, potentially explained by low gluconeogenic gene expression. (a) Cell number-based proliferation assays of HCC cell lines cultured in different concentrations of glucose. Data represent a single experiment with 3 biological replicates. (b) Cell number-based proliferation assays of HCC cell lines cultured in different concentrations of lipids (oleic acid). Data represent a single experiment with 3 biological replicates. (c) ATP-based cell proliferation assay of HCC cell lines. Data points represent the average of 6 biological replicates. (d) Mutation status of *TP53* and *CTNNB1* of HCC cell lines. (e) Simplified schematic of opposing glycolytic (red) and gluconeogenic (blue) pathways. G6PC: glucose-6-phosphatase, HX: hexokinase, G6P: glucose-6-phosphate, FBP1: fructose-1,6-bisphophatase 1, PFK: ATP-dependent 6-phosphofructokinase, F-1,6-BP: fructose-1,6-bisphophatase, PCK1: phosphoenolpyruvate carboxykinase (cytosolic), PK: pyruvate kinase. (f) qPCR mRNA expression of gluconeogenic genes in HCC cell lines compared to Primary Human Hepatocytes (PHH). Data represent a single experiment with 3 biological replicates (3 separate RNA samples). ****: *p*<0.0001, ordinary one-way ANOVA with Dunnett’s multiple comparisons test. (g) Normalized RNA-seq values for gluconeogenic genes in human HCC compared to normal liver. Data obtained from TCGA. ****: *p*<0.0001, *: *p*<0.05, unpaired two-tailed t test. *n* = 50(normal) and 374(tumor). All error bars: +/- SEM. (h) Kaplan-Meier plot of overall survival probability between low and high expression of gluconeogenic enzymes. Graphs were generated using the website: https://kmplot.com. **Supplementary Figure 2.** Constitutive GCGR expression in SNU398, but not other liver cancer cell lines, stimulates PKA activity in response to glucagon without inducing gluconeogenic gene expression. (a) Normalized RNA-seq values for glycolytic genes in human HCC compared to normal liver. Data obtained from TCGA. ****: *p*<0.0001, unpaired two-tailed t test. *n* = 50(normal) and 374(tumor). (b) qPCR mRNA expression of *GCGR* in Primary Human Hepatocytes (PHH) compared to immortalized, “normal” hepatocyte cell line THLE3. Data represent a single experiment with 3 biological replicates (3 separate RNA samples). (c) Quantification of cAMP in SNU398 expressing either eGFP or GCGR and treated with 100nM glucagon (100G). Each graph represents an independent experiment with 2 technical replicates. DMSO: dimethylsulfoxide, HOAc: 0.05M acetic acid, 20F: 20uM Forskolin (positive control), veh: vehicle (0.05M acetic acid), 20F: 20uM forskolin (positive control). (d) Long-term protein analysis of downstream effectors of cAMP signaling in SNU398 cells expressing either eGFP or GCGR and treated with 3 different concentrations of glucagon. Black triangles denote increasingly equivalent concentrations of vehicle compared to glucagon (red triangles). (e) qPCR mRNA expression of *G6PC* in SNU398 cells expressing either eGFP or GCGR and treated with 100nM glucagon (100G). Data represent a single experiment with 3 technical replicates (1 RNA sample). Error bars: +/- SD. veh: vehicle (0.05M acetic acid). (f) qPCR mRNA levels of *GCGR* in HCC cell lines compared to Primary Human Hepatocytes (PHH). Same pLenti-CMV-GCGR or pLenti-CMV-eGFP lentivirus as SNU398 was used for stable expression. Data represent a single experiment with 2 biological replicates (2 separate RNA samples). (g) Quantification of cAMP in HCC cell lines expressing either eGFP or GCGR and treated with 100nM glucagon (100G). Data points represent a single experiment of 2 technical replicates. veh: vehicle (0.05M acetic acid), 20F: 20uM forskolin (positive control). (h) Protein analysis of downstream effectors of cAMP signaling (PKA substrates) in HCC cell lines expressing GCGR and treated with either vehicle (0.05M acetic acid, (-)) or 100nM glucagon (+). (i) qPCR mRNA levels of *GCGR* and gluconeogenic enzyme genes in cryopreserved primary human hepatocytes (cPHH) treated with 100nM glucagon (100G). Data represent a single experiment with 2 biological replicates (2 separate RNA samples). (j) qPCR mRNA expression of *GCGR* in SNU398 cells expressing either eGFP or GCGR and treated with 100nM glucagon (100G). Data represent a single experiment with 3 biological replicates (3 separate RNA samples). Error bars: +/- SEM. veh: vehicle (0.05M acetic acid). ns: not significant, p>0.05, ordinary one-way ANOVA with Tukey’s multiple comparisons test. **Supplementary Figure 3.** Glucagon stimulation of gluconeogenic genes and treatment with epigenetic inhibitors across multiple liver cancer cell lines. (a) qPCR mRNA expression of *GCGR, G6PC, FBP1* and *PCK1* in HepG2, PLCPRF5 and Huh7 cells expressing either eGFP or GCGR and treated with 100nM glucagon (100G). Data represent a single experiment with 3 technical replicates (1 RNA sample). Error bars: +/- SD. veh: vehicle (0.05M acetic acid). (b) Protein analysis of EZH2 inhibitor efficacy in HepG2, PLC and Hep3B cells at the indicated drug concentrations, culture conditions, and time. (c) Protein analysis of EZH2 inhibitor efficacy in SNU398 cells at the indicated drug concentrations, culture conditions, and time. (d) Protein analysis of DNA methyltransferase inhibitor Decitabine efficacy in PLC cells compared to Primary Human Hepatocytes (PHH) at the indicated drug concentrations, culture conditions, and time. (e) Protein analysis of HDAC inhibitor efficacy in Hep3B, PLC and HepG2 cells at the indicated drug concentrations, culture conditions, and time. **Supplementary Figure 4.** Glucagon stimulation of *GCGR* and *FBP1* expression and treatment with epigenetic inhibitors across multiple liver cancer cell lines. (a) qPCR mRNA expression of *GCGR* in THLE3, Normal liver (*n*=4), Tumor tissue (*n*=4), HCC cell lines expressing eGFP, VAGA1 PDX tissue and PDX tissue (*n*=5) compared to Primary Human Hepatocytes (PHH). Data represent a single experiment with 3 biological replicates (3 separate RNA samples). ****: p<0.0001, ordinary one-way ANOVA with Dunnett’s multiple comparisons test. (b) qPCR mRNA levels of *GCGR* in M7571 cells treated with glucagon plus combinations of epigenetic drugs. Data represent a single experiment with 3 technical replicates (1 RNA sample). Error bars: +/- SD. (-): no drug, 100G: 100nM glucagon, vehicle: 0.035% of 0.05M acetic acid, EZH2i: 1uM GSK126, HDACi: 10nM LBH589, DNMTi: 5uM Decitabine, triple: 1uM GSK126 + 10nM LBH589 + 5uM Decitabine. (c) qPCR mRNA levels of *FBP1* in Hep3B, PLC and HepG2 cells treated with glucagon and/or combinations of epigenetic drugs. Data represent a single experiment with 3 technical replicates (1 RNA sample). Error bars: +/- SD. (-): DMSO, EZH2i: 10uM GSK126 (10G), HDACi: 10nM LBH589 (10L), DNMTi: 1uM Decitabine, triple: 10uM GSK126 + 10nM LBH589 + 1uM Decitabine. (d) ATP-based cell proliferation assay of HCC cell lines treated with combinations of epigenetic drugs. Data points represent the average of 3 biological replicates. Error bars: +/- SD. (-): DMSO, EZH2i: 10uM GSK126, HDACi: 10nM LBH589. **Supplementary Figure 5.** Epigenetic inhibitors reduce cell viability across multiple HCC cell lines but display high toxicity *in vivo*. (a) ATP-based cell viability assays performed on HCC cell lines treated with serially diluted (1:3) concentrations of epigenetic inhibitors. Data points represent the average of 6 biological replicates. Error bars: +/- SEM. DMSO used a vehicle control. Note, no maximal drug concentration included greater than 0.2% (1:500) of DMSO. Sorafenib used as a clinically relevant drug comparison. UNC0642 (G9a inhibitor (H3K9 dimethylation)) used as an emerging epigenetic target comparison. (b) Crystal violet assay on SNU398 cells treated with EZH2 and pan-HDAC inhibitors. Each well represents a biological replicate. (c) Tumor volume measurements of SNU398 xenografts in Nu/J mice treated with EZH2 (GSK126) and pan-HDAC (LBH589) inhibitors at the indicated dosage. Treatments were performed at irregular intervals (due to loss/recovery of weight) over the course of the experiment by intraperitoneal injection. vehicle: 20% 2-HP-B-CD (hydroxypropyl-beta-cyclodextrin), pH 4.5. N=5 mice per treatment cohort with 2 tumors per mouse. Data points represent average volume of 10 tumors. Error bars: +/- SEM. (d) Body weight measurements of mice treated with EZH2 (GSK126) and pan-HDAC (LBH589) inhibitors at the indicated dosage in same experiment as (c). Data points represent average weights of 5 mice. Error bars: +/- SEM. **Supplementary Figure 6.** Glucagon/GCGR only decreases cell viability in SNU398 through an unknown mechanism independent of CREB. (a) Cell proliferation assays on liver cancer cell lines either expressing eGFP or GCGR and treated with 100nM glucagon. Data points represent 3 biological replicates. Error bars: +/- SEM. (b) Crystal violet assay on SNU398 cells either expressing eGFP or GCGR, treated with 100nM glucagon (100G), and transfected with 25nM of a small interfering RNA molecular targeting CREB1 (siCREB). Cells were initially treated with glucagon for 3 days and then transfected with siCREB without any further treatment. siNTC (25nM): non-targeting control, veh: vehicle (0.05M acetic acid). (c) Protein analysis of siRNA efficacy in SNU398 cells either expressing eGFP or GCGR and treated with 100nM glucagon. Samples harvested at the 7-day time point as illustrated in the previous figure panel. siCycloB (25nM): Cyclophilin B (positive control for transfection protocol). (d) Protein assessment of the target efficacy of CREB antagonist, 666-15, in SNU398 cells either expressing eGFP or GCGR and treated with or without 100nM glucagon. (e) PI/Annexin V flow cytometry analysis of SNU398 cells expressing either eGFP or GCGR and treated with either 100nM glucagon, 0.5uM 666-15, or the combination. Data points represent average of 3 biological replicates. Error bars: +/- SEM. 10ug/ml blasticidin used as a positive control. ns: not significant, **: adjusted *p*=0.0055, ordinary one-way ANOVA with Tukey’s multiple comparisons test.

## Data Availability

All raw data and/or reagents from this study are available from the corresponding author on request.

## Abbreviations

GCGR: G-coupled glucagon receptor
G6PC: Glucose-6-phosphatase catalytic subunit
FBP1: Fructose-1,6-bisphosphatase 1
PCK1: Phosphoenolpyruvate carboxykinase 1
cAMP: Cyclic adenosine monophosphate
PKA: Protein kinase A
CREB: cAMP response element binding protein
CAMKII: Calcium/calmodulin-dependent protein kinase II
PHH: Primary human hepatocytes
TCA: Tricarboxylic acid cycle
EZH2: Enhancer of zeste homolog 2
HDAC: Histone deacetylase
DNMT: DNA methyltransferase
DMSO: Dimethylsulfoxide
CoA: Coenzyme A
eGFP: Enhanced green fluorescent protein
