# Supplementary material for: Glucagon signaling via supraphysiologic GCGR can reduce cell viability without stimulating gluconeogenic gene expression in liver cancer cells
Source: Cancer Metab. 2022 Feb 5;10:4. doi: 10.1186/s40170-022-00280-1 (PMC8817478; doi:10.1186/s40170-022-00280-1)
Supplement: Supplementary file 1 — Additional file 1: Supplementary Figure 1. Liver cancer cells display hypersensitivity to long- and short-term glucose and lipid withdrawal, potentially explained by low gluconeogenic gene expression. (a) Cell number-based proliferation assays of HCC cell lines cultured in different concentrations of glucose. Data represent a single experiment with 3 biological replicates. (b) Cell number-based proliferation assays of HCC cell lines cultured in different concentrations of lipids (oleic acid). Data represent a single experiment with 3 biological replicates. (c) ATP-based cell proliferation assay of HCC cell lines. Data points represent the average of 6 biological replicates. (d) Mutation status of TP53 and CTNNB1 of HCC cell lines. (e) Simplified schematic of opposing glycolytic (red) and gluconeogenic (blue) pathways. G6PC: glucose-6-phosphatase, HX: hexokinase, G6P: glucose-6-phosphate, FBP1: fructose-1,6-bisphophatase 1, PFK: ATP-dependent 6-phosphofructokinase, F-1,6-BP: fructose-1,6-bisphophatase, PCK1: phosphoenolpyruvate carboxykinase (cytosolic), PK: pyruvate kinase. (f) qPCR mRNA expression of gluconeogenic genes in HCC cell lines compared to Primary Human Hepatocytes (PHH). Data represent a single experiment with 3 biological replicates (3 separate RNA samples). ****: p<0.0001, ordinary one-way ANOVA with Dunnett’s multiple comparisons test. (g) Normalized RNA-seq values for gluconeogenic genes in human HCC compared to normal liver. Data obtained from TCGA. ****: p<0.0001, *: p<0.05, unpaired two-tailed t test. n = 50(normal) and 374(tumor). All error bars: +/- SEM. (h) Kaplan-Meier plot of overall survival probability between low and high expression of gluconeogenic enzymes. Graphs were generated using the website: https://kmplot.com. Supplementary Figure 2. Constitutive GCGR expression in SNU398, but not other liver cancer cell lines, stimulates PKA activity in response to glucagon without inducing gluconeogenic gene expression. (a) Normalized RNA-seq val [file 40170_2022_280_MOESM1_ESM.pdf]

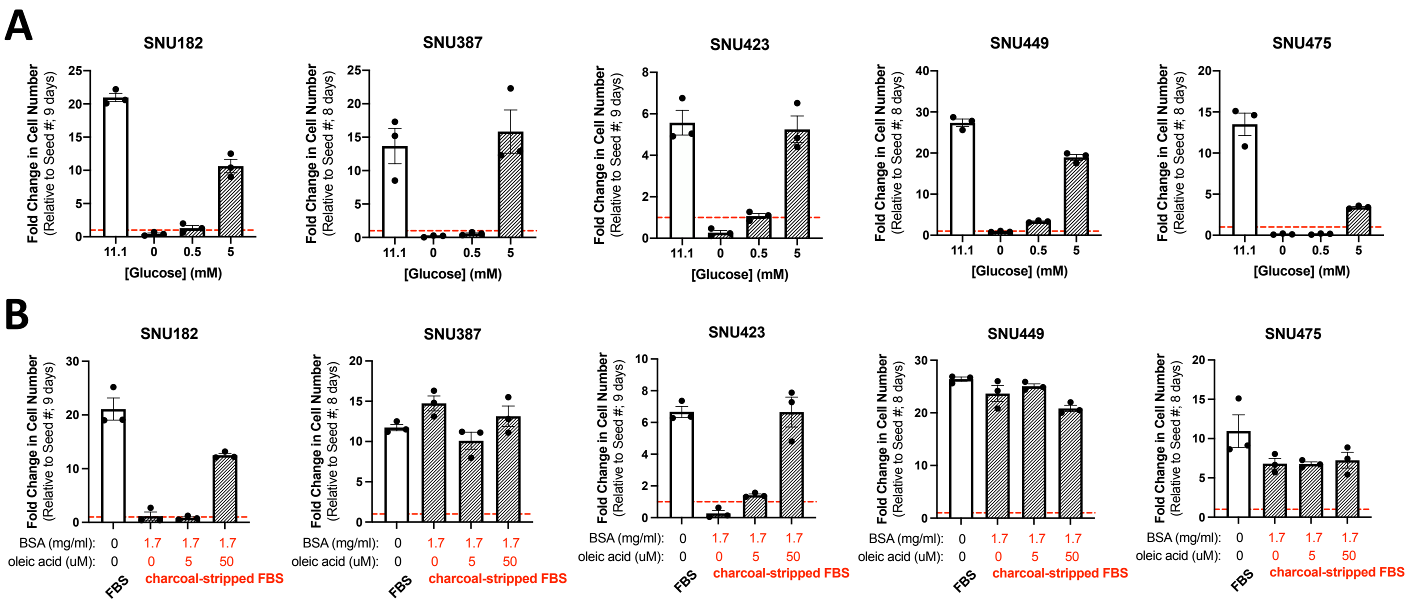

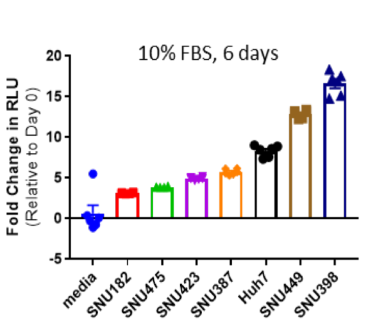

D

Cell Lines	SNU182	SNU397	SNU398	SNU423	SNU449	SNU475	Hep3B	PLC	HepG2	SK-HEP1	Huh7
TP53	S215I	K164*	splice	splice	A161T	N239D	WT	R249S	WT	WT	Y220C
CTNNB1	WT	WT	R249S	WT	WT	WT	WT	WT	WT	WT	WT

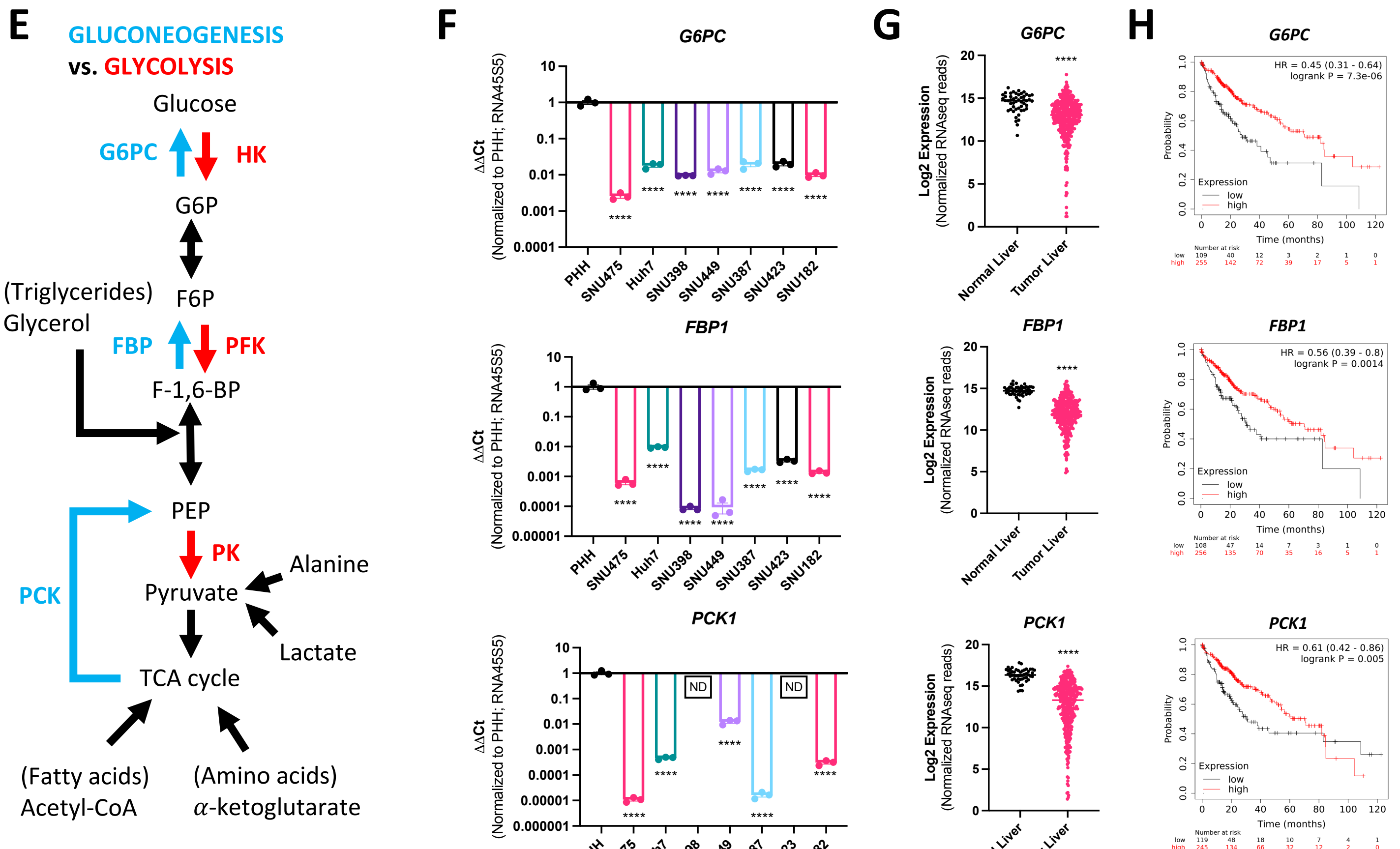

Supplementary Figure 1

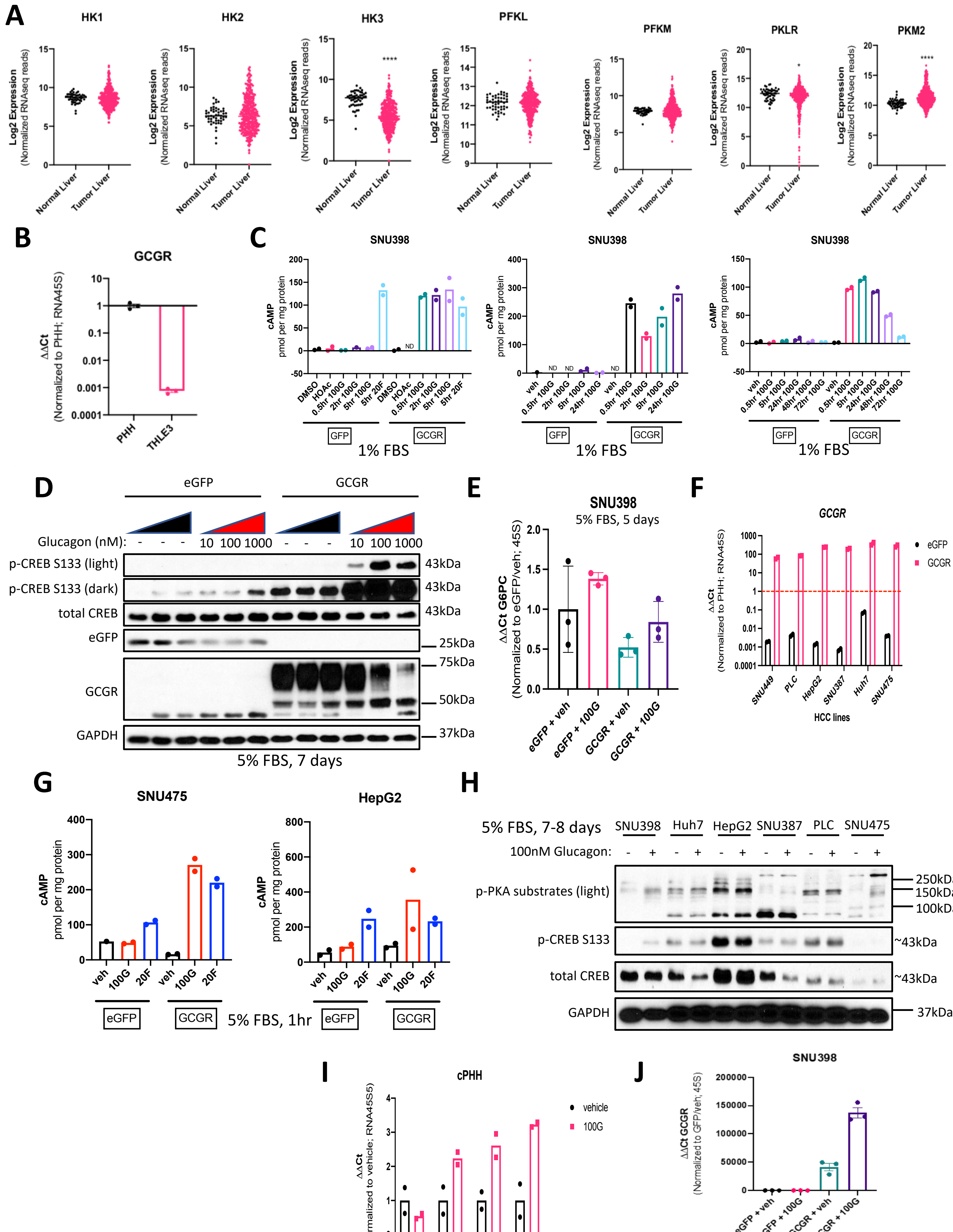

Supplementary Figure 2

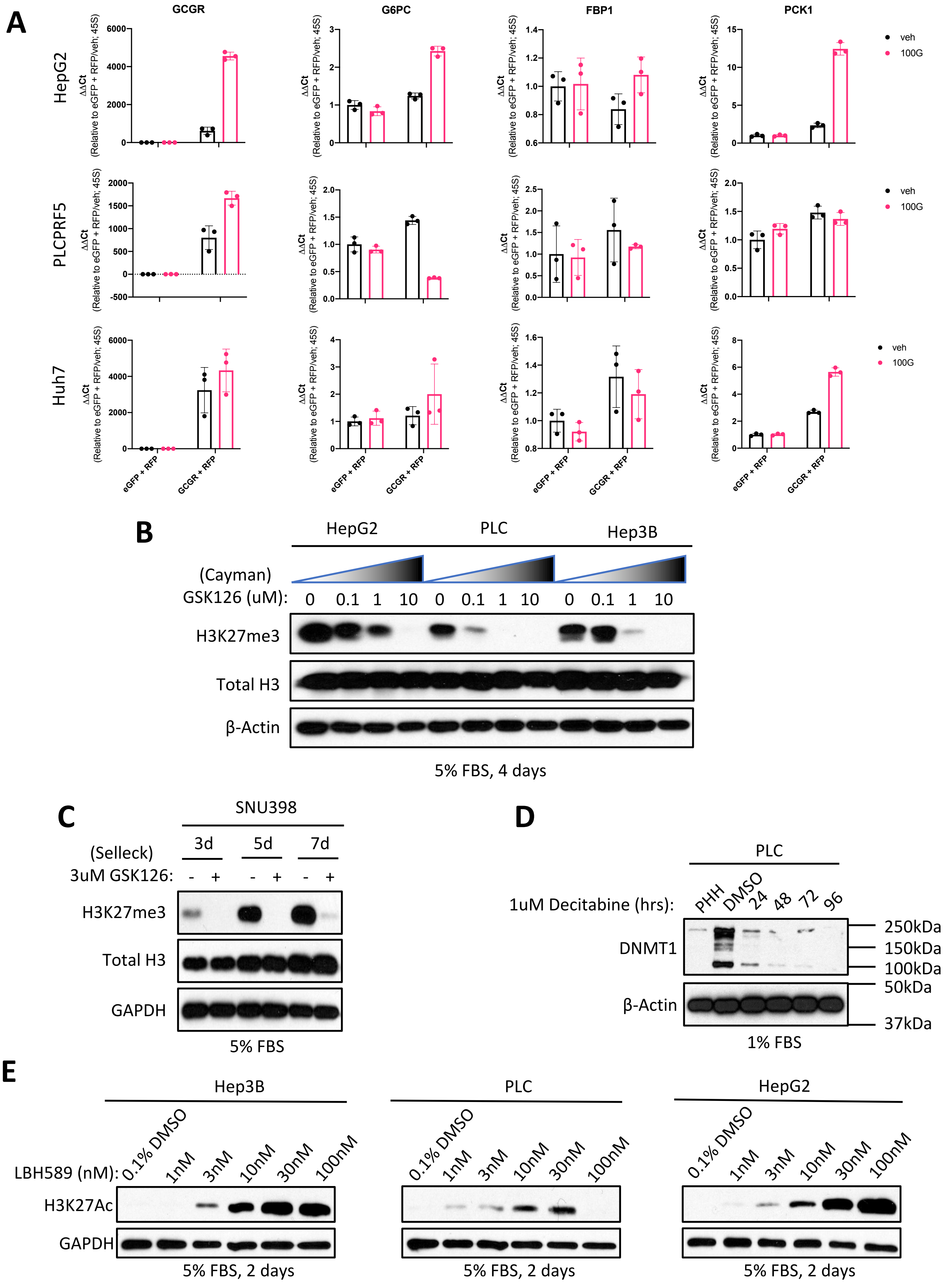

Supplementary Figure 3

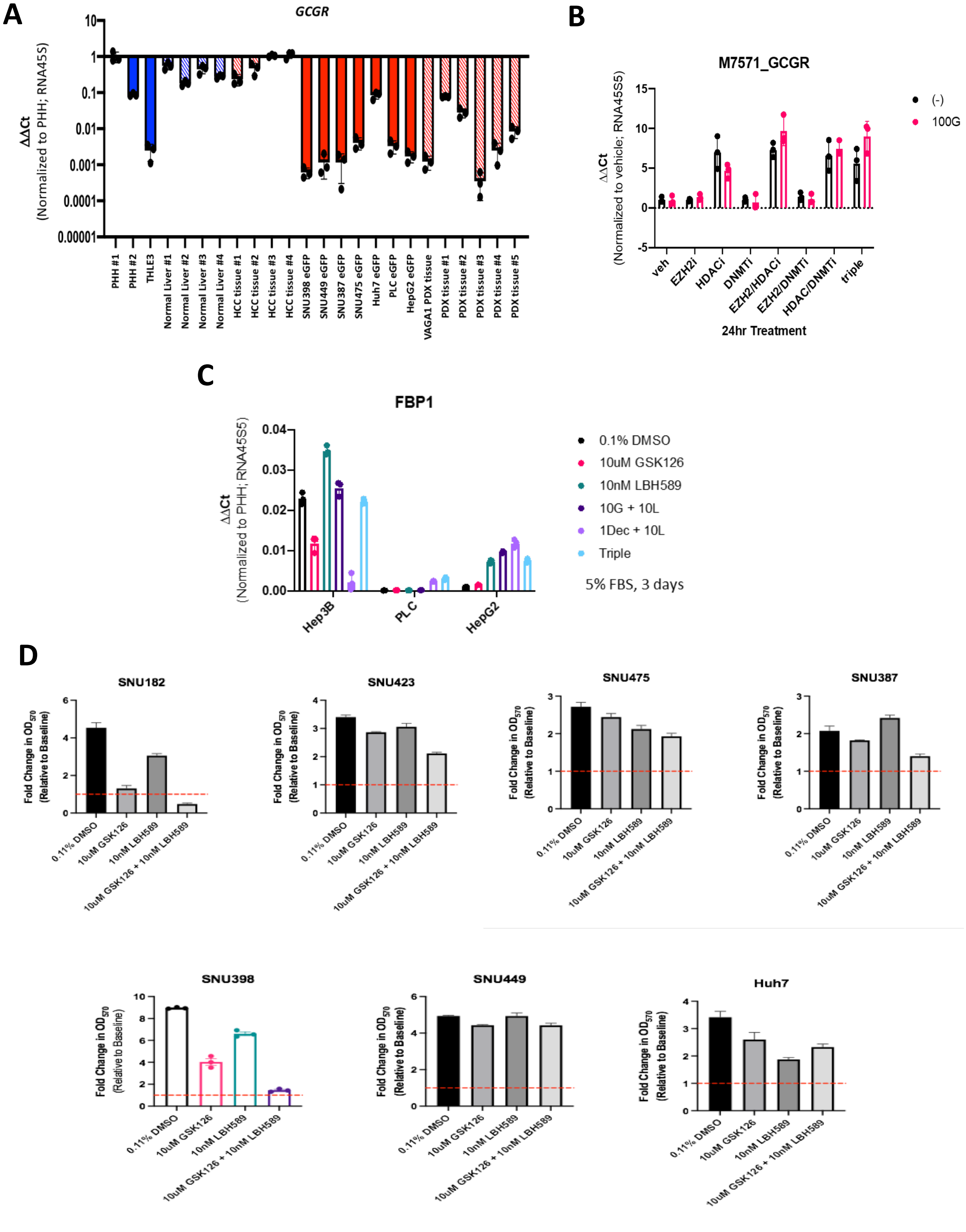

Supplementary Figure 4

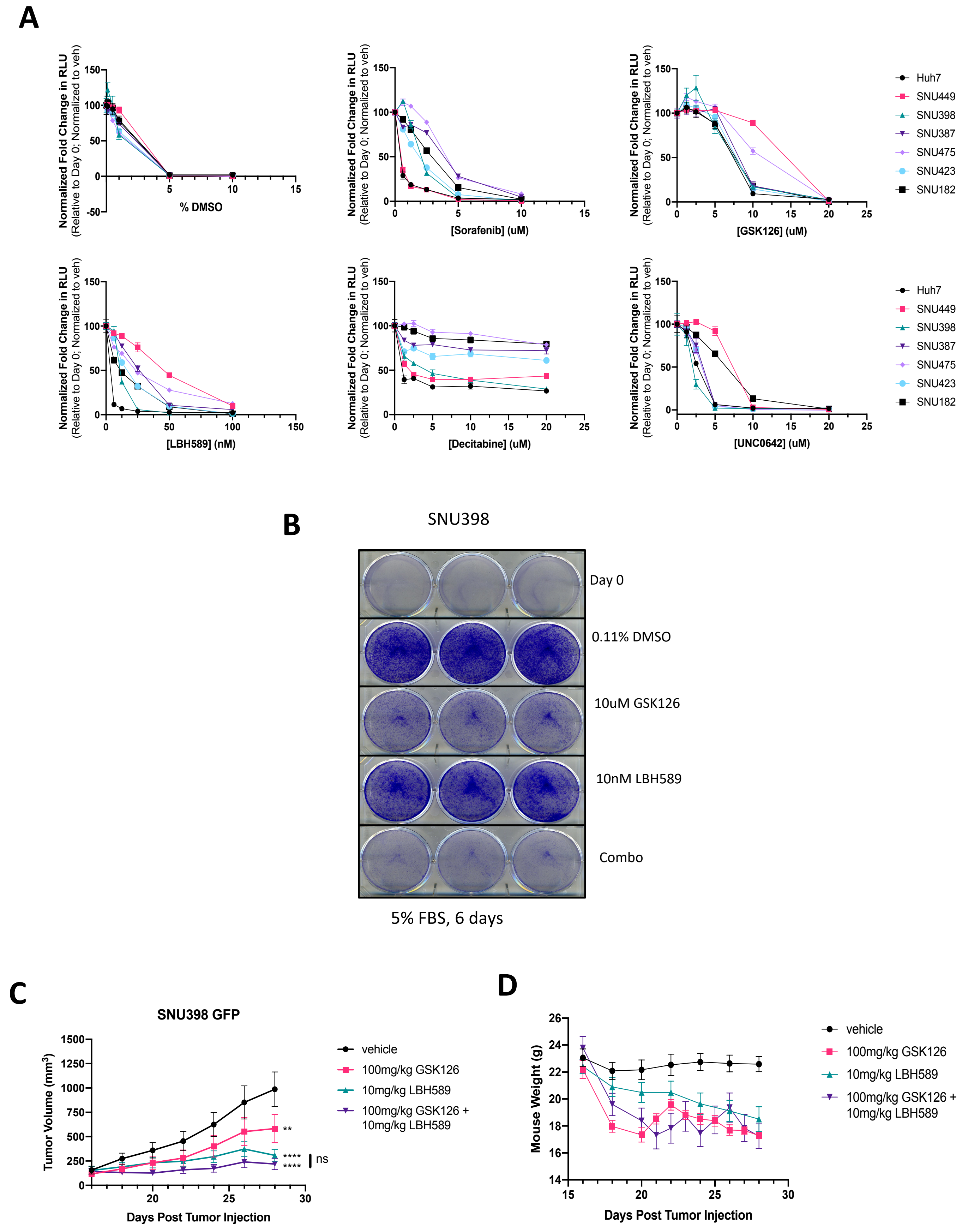

Supplementary Figure 5

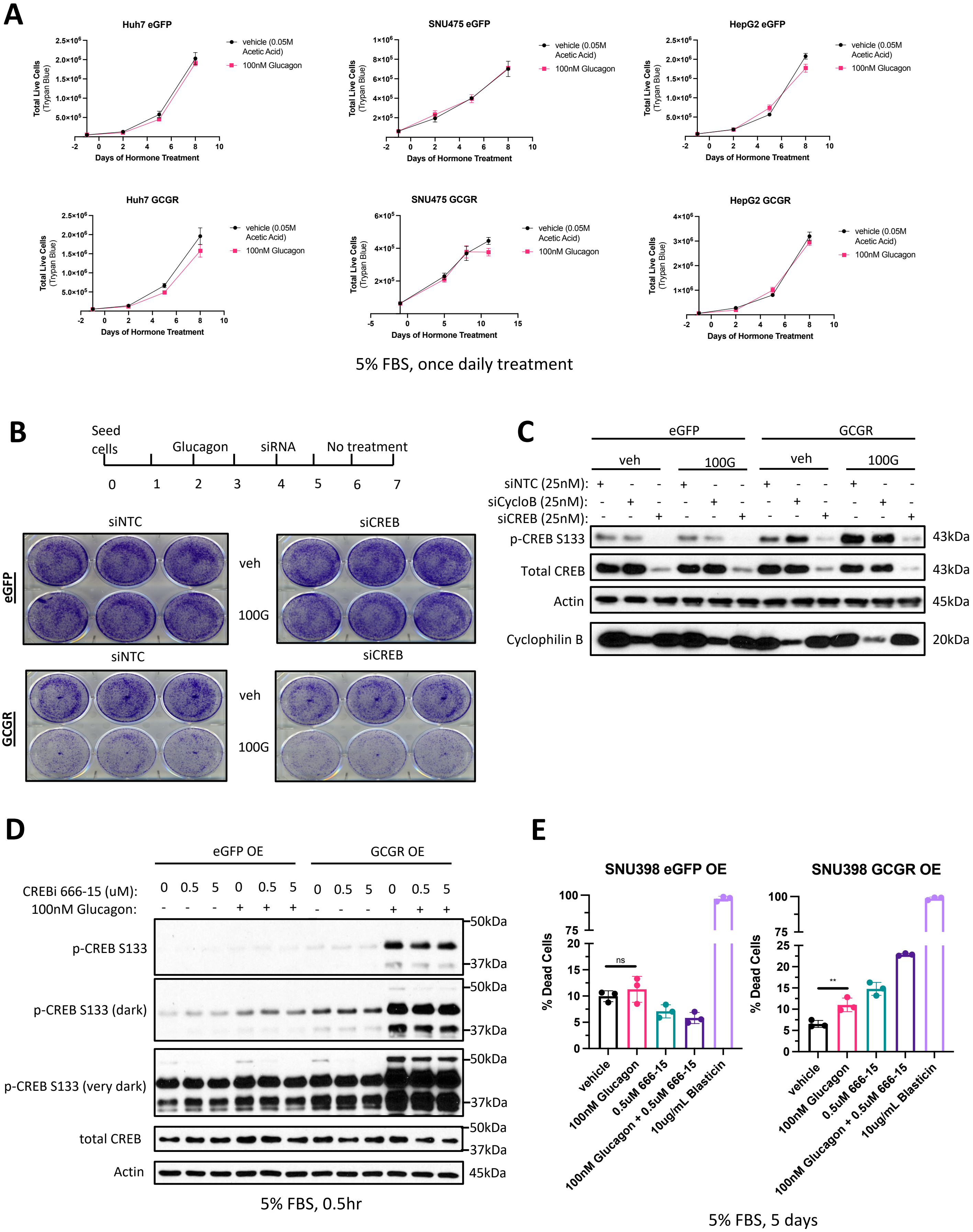

Supplementary Figure 6
